# Supplementary material for: Alteration of Membrane Physicochemical Properties by Two Factors for Membrane Protein Integration
Source: Biophys J. 2019 May 21;117(1):99–110. doi: 10.1016/j.bpj.2019.05.014 (PMC6626835; doi:10.1016/j.bpj.2019.05.014)
Supplement: Document S1. Supporting Methods, Figs. S1–S6, and Tables S1–S4 [file mmc1.pdf]

Biophysical Journal, Volume *117*

Supplemental Information

Alteration of Membrane Physicochemical Properties by Two Factors for Membrane Protein Integration

Kaoru Nomura, Toshiyuki Yamaguchi, Shoko Mori, Kohki Fujikawa, Ken-ichi Nishiyama, Toshinori Shimanouchi, Yasushi Tanimoto, Kenichi Morigaki, and Keiko Shimamoto

Supplemental Information

Alteration of Membrane Physicochemical Properties by Two Factors for Membrane Protein Integration

Kaoru Nomura, Toshiyuki Yamaguchi, Shoko Mori, Kohki Fujikawa, Ken-ichi Nishiyama, Toshinori Shimanouchi, Yasushi Tanimoto, Kenichi Morigaki, and Keiko Shimamoto

METHODS

Solid-state NMR measurement

Sample preparation

Multilamellar vesicles (MLVs) of *E. coli* polar lipid (EPL) for NMR measurements (Fig. 2a, 3 and 4b) were prepared as described previously (1-3). Designated amounts of natural MPIase, mini-MPIae-3, and/or DAG were co-solubilized with EPL in chloroform/methanol and the mixture was dried under nitrogen gas stream. After solvent evaporation under vacuum overnight, the resulting lipid film was hydrated with Tris buffer (20 mM Tris-HCl, 150 mM NaCl, pH 7.5) and vortex mixed. The suspension was freeze-thawed for ten cycles and lightly sonicated. The suspension was packed within a 4 mm NMR tube closed with the sealing-equipped cap to prevent drying (Phi Creative, Kyoto, Japan). For ^{2}H NMR measurements, we added ^{2}H-labeled DMPE (5 wt%) into the EPL liposomes. So as to mimic the molar ratio of *E. coli* membrane lipid components (DMPE/DMPG = 3/1 by weight), non-labeled DMPG (1.7 wt%) was also added. Deuterium-depleted water was used to prepare the

2-[4-(2-hydroxyethyl)piperazin-1-yl]ethanesulfonic acid (HEPES) buffer.

General measurement of solid-state NMR

All solid-state NMR measurements were carried out using a Bruker Avance III 600 spectrometer (Bruker Biospin, AG, Switzerland) equipped with a narrow-bore magnet operated at a resonance frequency of 150.13 MHz for ^{13}C, 242.94 MHz for ^{31}P, 92.12 MHz for ^{2}H, and 600.13 MHz for ^{1}H. Data were recorded using a cross polarization (CP) magic angle spinning (MAS) probe and an E-free triple-resonance probe. The MAS rate was 5 kHz for the measurement of ^{13}C CP and direct polarization (DP) MAS spectra (Fig. 3a), and ^{31}P relaxation time experiments (Fig. 3b and c). ^{31}P NMR spectra (Fig. 2a) and ^{2}H spectra (Fig. 4b) were acquired without spinning. Typical 90° pulse lengths for ^{13}C, ^{31}P, ^{2}H, and ^{1}H were 4.4, 5.0, 4.5, and 4.5 μs respectively. ^{1}H-^{13}C CP measurements were performed using a ramped (from 50% to 100%) spin-lock pulse on the ^{1}H channel and a square contact pulse on the ^{13}C channel with a 2 ms contact time. During acquisition, 51 kHz SPINAL-64 ^{1}H decoupling (4) was performed for all experiments. The ^{31}P and ^{13}C chemical shifts were externally referenced to 85% H_3PO_4 (0 ppm) and the methylene carbon of adamantane (40.48 ppm), respectively (5).

Quadrupole splitting

^{2}H spectra were obtained by use of the quadrupolar echo sequence (6) with an echo delay of 30 μs, and a recycling delay of 500 ms. The number of accumulated scans varied from 500,000 to 1000,000. All ^{2}H were processed using 500 Hz line broadening. The quadrupole splitting in a ^{2}H NMR spectra is given by the equation:

$$\Delta\nu = 3/4(e^2Qq/h)(3\cos^2\gamma\text{-}1)S_{CD} \qquad \text{(S1)},$$

where (e^2Qq/h) is the quadrupole coupling constant and γ is the angle between lipid long axis and magnetic field (6). As shown in Fig. 2a, liposome was magnetically aligned in each sample, γ was almost 90°. S_{CD} is the order parameter of CD bond (C4-^{2}H4) vector against the lipid long axis direction.

^{31}P T_1 relaxation times

^{31}P T_1 relaxation times were measured using the standard inversion recovery sequence (180°-τ-90°) (7). Delay τ varied between 1 ms and 2 s. The intensities were fit to a single-exponential function, $I = 1\text{-}2\exp(-\tau/T_1.)$.

Liposome observation

Sample preparation

Large unilamellar vesicles (LUVs) used for observing liposomes by transmission electron microscope (TEM) and dynamic light scattering (DLS) (Fig. S3) and analyzing the percentage of membrane fusion (Fig. 2b) were prepared with HEPES buffer (50 mM HEPES-KOH, 150 mM NaCl, pH 7.0) as described previously (8). Four types of LUVs composed of EPL, EPL/DAG (5 wt%), EPL/MPIase (5 wt%) or EPL/DAG (5 wt%)/MPIase (5 wt%) were prepared at a final EPL lipid concentration of 0.2 mg/ml and then transformed to 100 nm LUVs using an extruder (Avestin, Ottawa, Canada).

TEM observation

Liposomes were observed using a JEOL JEM-3100FEF transmission electron microscope (JEOL, Tokyo, Japan) with an acceleration voltage of 300 kV. An aliquot of the sample was poured onto the cell and rapidly frozen using an EM-CPC (LEICA, Wetzlar, Germany). The frozen samples were thereafter loaded into the instrument to observe the cryo-TEM image.

DLS measurement

DLS measurements were carried out at 30°C using a Nano Particle Size Analyzer (SZ-100, Horiba Scientific, Japan) with a 10 mW He-Ne laser emitting at 532 nm. Samples (600 μl) were placed in disposable polystyrene cuvettes with a 1.0-cm path length. Scattered light was detected at 90° of the incident beam using an APD detector. The hydrodynamic radius values were acquired using standard mode and calculated using the Stokes-Einstein equation.

Cobalt-calcein method

The percentage of fusion of liposome membranes was measured using the cobalt (Co^{2+})-calcein method (9). The Co^{2+}-calcein (100 mM)-containing and 2,2',2'',2'''-(ethane-1,2-diyldinitrilo)tetraacetic acid (EDTA) (100 mM)-containing liposomes were mixed at a ratio of 1:1 in 1 mM cobalt citrate solution. The fusion reaction was performed by incubation of the sample at 30 or 37 °C for 20 min. When the liposomes were fused, the fluorophore was liberated from the quenching mode by chelation of Co^{2+} with EDTA. The fluorescence intensity induced by free calcein within the fused liposomes was measured at an excitation wavelength of 490 nm and an emission wavelength of 520 nm. Fluorescence due to the leaking of liposome content was immediately quenched by cobalt citrate in the bulk solution. Without EDTA, neither the Co^{2+}-calcein complex nor calcein-citrate complex showed fluorescence. The percentage of membrane fusion was calculated from the equation:

$$\text{Fusion (\%)} = 100 * I_m/I_t, \qquad \text{(S2)}$$

where I_m is the measured fluorescence intensity of the sample. The total fluorescence, I_t,

was determined after the disruption of the liposome membrane by the addition of Triton X-100 at a final concentration of 2 mM in the absence of cobalt citrate solution.

Membrane packing measurement

To prepare LUVs for lipid packing analyses (Fig. 4c and d), laurdan was added to the lipid mixture at a molar ratio of 1:100. Fluorescence spectra were acquired on a F-4500 fluorescence spectrophotometer (HITACHI High-Technologies Corp., Tokyo, Japan). The emission spectra were measured from 400 to 600 nm with an excitation wavelength of 360 nm and a 5 nm bandwidth at 30 °C. The background measured in HEPES buffer was subtracted from all emission spectra. Laurdan generalized polarization (GP) was calculated using the following equation:

$$GP = (I_{440} - I_{490})/(I_{440} + I_{490}), \quad \text{(S3)}$$

where I_{440} and I_{490} are the intensities at 440 and 490 nm of the emission spectrum, respectively (10).

Flip-flop assay

To obtain 1-palmitoyl-2-{12-[(7-nitro-2-1,3-benzoxadiazol-4-yl)amino]dodecanoyl}-sn-glycerol (C_{12}-NBD-DAG), 1 mg of 1-palmitoyl-2-{12-[(7-nitro-2-1,3-Benzoxadiazol -4-yl)amino]dodecanoyl}-sn-glycero-3-phosphocholine (C_{12}-NBD-PC) (was digested for 2 h at 37 °C using *C. welchii*-phospholipase C (10 U) in 3.0 ml of diethyl ether/95% ethanol/water (49:1:50, v/v/v) with 0.6 μM $CaCl_2$ under shaking. Then, the produced C_{12}-NBD-DAG in the upper phase was extracted and washed with the mixture of

chloroform/water (1:1, v/v) twice. The purity of C_{12}-NBD-DAG was checked by thin-layer chromatography (TLC) and NMR. The weight of C_{12}-NBD-DAG was measured with an Ultra-Microbalance XP6V (Mettler-Toledo, Greifensee, Switzerland).

A designated amount of mini-MPIase-3 was solubilized with EPL in chloroform. C_{12}-NBD-DAG was added to EPL at a weight ratio of 1:100 and then the mixture was dried completely. The lipid film was hydrated with HEPES buffer for an EPL concentration of 0.1 mM. The suspension was freeze-thawed for ten cycles and extruded through a 100-nm pore filter (Avestin) to give LUV. To evaluate the effects of mni-MPIase-3 on the dithionite quenching in the outer leaflet, we prepared the C_{12}-NBD-PC containing liposomes in the same manner.

After monitoring the fluorescence of 350 μl LUV (100 μM) without dithionite for more than 10 s on an F-4500 fluorescence spectrophotometer (HITACHI High-Technologies Corp.) at excitation and emission wavelengths of 475 and 530 nm, respectively, 50 μl of 100 mM sodium dithionite (final concentration 12.5 mM) was added to the LUV suspension (final LUV concentration 87.5 μM). The time course of the fluorescence decrease by dithionite was continuously monitored. The flip-flop time constant (t_{flop}) was calculated from the dithionite quenching profile (11-13). The normalized fluorescence intensities F(t)/F(0), where F(0) is the fluorescence intensity just before adding dithionite, is shown by the following equation (12):

$$F(t)/F(0) = A_{inner} \exp(-t/t_{flop}) + A_{outer} \exp(-t/t_q) + C \quad \text{(S4)}$$

where A_{inner} and A_{outer} are the molar fraction of the fluorescent lipids in the inner and outer leaflets before adding dithionite, and t_{flop} and t_q are the time constants of the

flip-flop movement and dithionite quenching of C_{12}-NBD-DAG in the outer leaflet, respectively. C is the fluorescence intensity where the fluorescence attained a plateau. Usually, fluorescence was not completely quenched because some vesicles still formed multilamellar structures.

Formation of supported planar lipid bilayers

Small unilamellar vesicles (SUVs) used for supported planar lipid bilayer (SPB) observations (Fig. 6) were prepared as described previously (3). Four types of SUVs composed of EPL, EPL/DAG (5 wt%), EPL/DAG (5 wt%)/MPIase (5 wt%), and EPL/DAG (5 wt%)/MPIase (20 wt%) were prepared at a final EPL lipid concentration of 1 mg/ml in phosphate buffer (10 mM Na_2HPO_4, 150 mM NaCl, pH 6.6). Finally, the lipid dispersions were sonicated in ice water for 3-5 min using a Branson SFX150 Sonifier (Branson Ultrasonics, Danbury, CT) at 5 W. For observation of SPBs, 0.01 mg/ml of TR-DHPE was added to the EPL SUVs as a component of the lipid mixtures.

Glass slides were cleaned first by sonicating in 0.1 M SDS solution for 20 min and rinsing with deionized water. Then, they were treated in a cleaning solution of 0.05:1:5 NH_4OH (28%)/H_2O_2 (30%)/H_2O for 10 min at 65 °C and again rinsed extensively with deionized water, and then dried in a vacuum oven for 30 min at 80 °C. After cleaning by exposure to UV-produced ozone for 30 min, a SUV suspension was deposited onto a glass slide. The vesicles were allowed to adsorb and form SPBs on the surface at room temperature. SPBs thus formed were subsequently rinsed with the HEPES buffer.

Fluorescence images of SPBs were acquired with a BX51WI microscope (Olympus, Tokyo, Japan) equipped with a water immersion objective lens (60x, N.A. = 0.9, Olympus) and a 75 W Xenon lamp (LH75XEAPO). WIY filters (Olympus) were

used to detect Texas-Red fluorescence. Images of SPB fluorescence were captured with a charge coupled device (CCD) camera (DP30BW, Olympus) mounted on the microscope and further processed with Metamorph (version 6.3, Molecular Devices, CA, U.S.A.) software.

Lateral diffusion measurement by the boundary profile evolution method

Lateral diffusion coefficients of the bilayers with/without DAG and MPIase were determined by the boundary profile evolution (BPE) method (14, 15) as described previously (3). After partially photo bleaching TR-PE in the SPBs by intense illumination for 15 s, the fluorescence recovery was monitored by obtaining images every 10 s. The intensity profiles perpendicular to the boundary were nonlinearly fitted to the following Gaussian error function:

$$2\frac{F(x,t)-F_{\text{bleached}}}{F_{\text{unbleached}}-F_{\text{bleached}}}=\operatorname{erf}\left(\frac{x-x_{\text{b}}}{2w}\right)+1, \quad \text{(S5)}$$

where $F(x, t)$ is the profile evolution with time, F_{bleached} and $F_{\text{unbleached}}$ are the fluorescence intensities inside and outside of the bleached region immediately after the bleaching, respectively, x_b is the position of the boundary between the bleached and unbleached areas, and $(x\text{-}x_b)$ is the distance to this boundary. The diffusion depth w is defined as

$$w=\sqrt{Dt}\,, \quad \text{(S6)}$$

where D is the diffusion coefficient and t is the elapsed time after bleaching. All measurements were conducted at room temperature.

TABLES

Table S1. Average fatty acid composition of *E.coli* phosphatidylethanolamine (PE), phosphatidylglycerol (PG), and cardiolipin(CL) in *E.coli* polar extract (16). Each component in EPL contains several types of fatty acids.

E.coli PE	
fatty acid	% of total fatty acid
18:1	34.1
16:0	33.6
17:0	17.7
16:1	9.3
19:0	3.8
14:0	1.5

E.coli PG	
fatty acid	% of total fatty acid
16:0	43.6
cyclo 17:0	26.8
Trans 18:1	9.7
16:1	7.4
cyclo 19:0	5.6
14:0	1.6
15:0	1.2
17:0	0.5
18:0	0.4
cis 18:0	0.1
unknown	3.2

E. coli CL	
fatty acid	% of total fatty acid
16:0	33.3
cyclo 17:0	27.0
trans 18:1	14.4
16:1	10.2
19:0	5.3
15:0	4.8
14:0	2.2
17:0	1.4
unknown	1.4

Table S2. Time constant of C_{12}-NBD-DAG flip-flop movement, t_{flop}, and dithionite quenching of C_{12}-NBD-DAG in the outer leaflet, t_q, in EPL/C_{12}-NBD-DAG (100:1 w/w) liposomes with various amounts of MPIase at 30 °C.

MPIase conc. (wt%)	t_{flop} (sec)	t_q(sec)
0	18.77 ± 0.03	1.74 ± 0.02
5	23.49 ± 0.04	1.76 ± 0.02
20	33.49 ± 0.10	3.26 ± 0.00
50	48.63 ± 0.22	4.44 ± 0.06

Normalized fluorescence decay curve shown in Figure 5a were fitted with the double exponential decay equation, $F(t)/F(0) = A_{inner} \exp(-t/t_{flop}) + A_{outer} \exp(-t/t_q) + C$, in Eq. S4.

Table S3. Time constant of C_{12}-NBD-DAG flip-flop movement, t_{flop}, and dithionite quenching of C_{12}-NBD-DAG in the outer leaflet, t_q, in EPL/C_{12}-NBD-DAG (100:1 w/w) liposomes with various amounts of NaCl in the presence and absence of MPIase (20 wt%) at 30 °C.

NaCl conc. (mM)	MPIase conc. (wt%)	t_{flop} (sec)	t_q(sec)
150	20	33.49 ± 0.10	3.26 ± 0.00
	0	18.77 ± 0.03	1.74 ± 0.02
300	20	28.41 ± 0.08	2.26 ± 0.03
	0	17.89 ± 0.02	1.41 ± 0.01
500	20	21.27 ± 0.03	1.51 ± 0.01
	0	18.60 ± 0.04	1.34 ± 0.01

Normalized fluorescence decay curve shown in Figure 5b were fitted with the double exponential decay equation, $F(t)/F(0) = A_{inner} \exp(-t/t_{flop}) + A_{outer} \exp(-t/t_q) + C$, in Eq. S4.

Table S4. Time constant of dithionite quenching of C_{12}-NBD-PC in the outer leaflet, t_q, in EPL/C_{12}-NBD-PC (100:1 w/w) liposomes with various amounts of MPIase at 30 °C.

MPIase conc. (wt%)	t_q(sec)
0	10.86 ± 0.05
5	11.49 ± 0.04
20	22.13 ± 0.04

Normalized fluorescence decay curve shown were fitted with the single exponential decay equation, $F(t)/F(0) = A_{outer} \exp(-t/t_q) + C$.

DATA GRAPHS

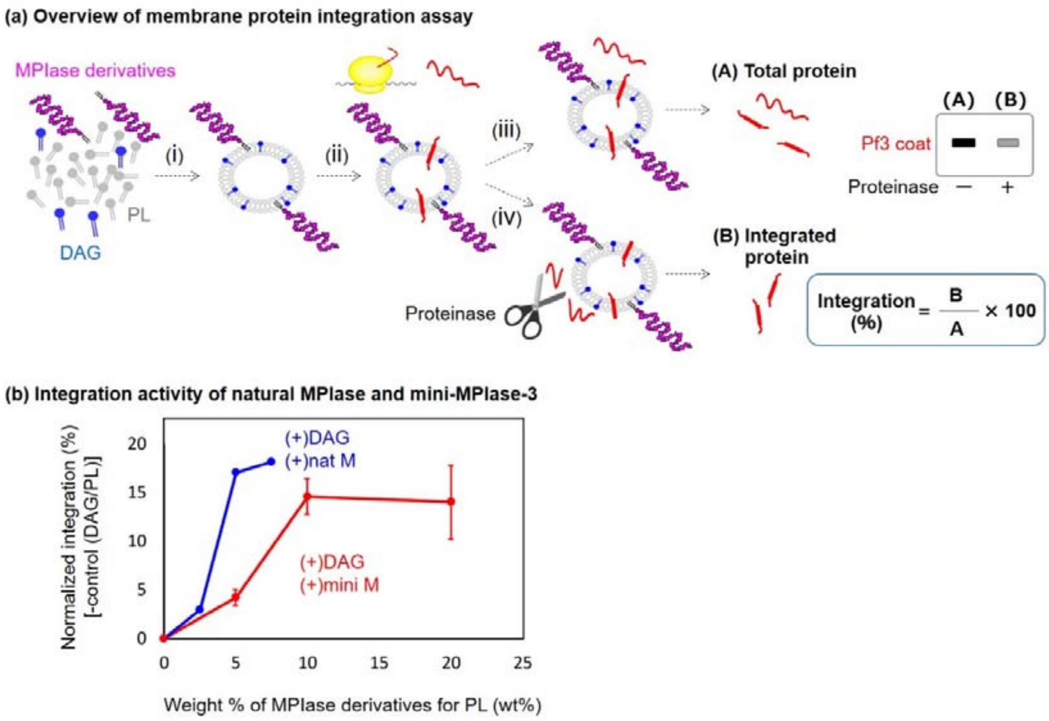

Figure S1. (a) Overview of the membrane protein integration activity assay. (i) Liposomes are prepared from DAG (5 wt%), *E. coli* polar lipids (EPL) and MPIase derivatives. (ii) Radioisotope-labeled 3L-Pf3 proteins are synthesized using an *in vitro* translation system in the presence of liposomes. (iii) The reaction mixture is divided into two fractions: One fraction gives the total amount of synthesized proteins by autoradiography after SDS-PAGE (band A). (iv) When protease is added to the other fraction, proteins outside liposomes are cleaved; thus, band B reflects the amount of proteins integrated in the liposomes. (b) Dose-response of the integration activity by MPIase derivatives. Normalized integration values were calculated by the subtraction of the percentage of control liposomes containing only DAG from the net integration percentages of liposomes containing MPIase derivatives.

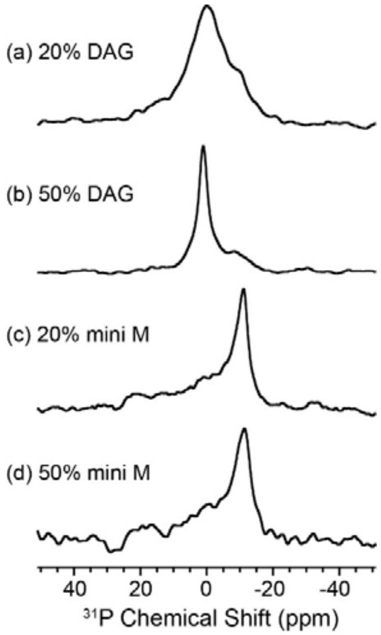

Figure S2. ^{31}P NMR spectra of *E. coli* polar lipid (EPL) in the presence of 20 and 50 wt% DAG (a and b) or 20 and 50 wt% mini-MPIase-3 (c and d). The amounts of DAG and mini-MPIase-3 are shown against the amount of EPL.

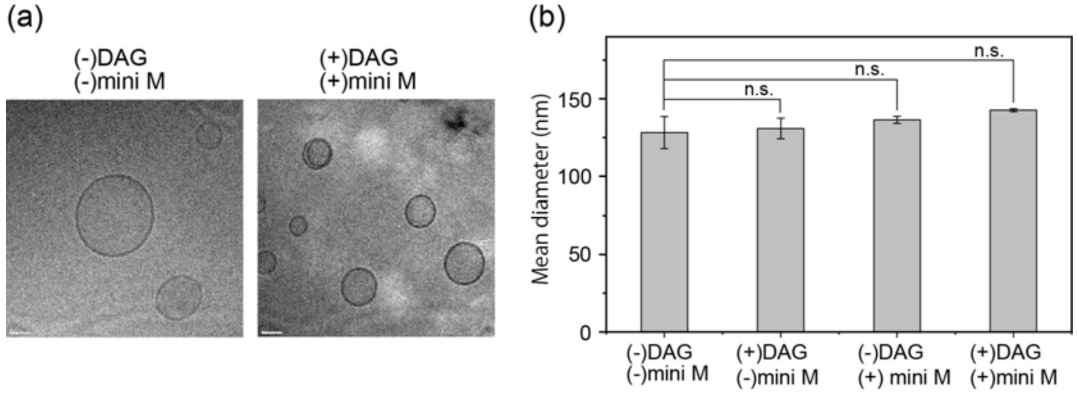

Figure S3. TEM image of EPL liposomes in the absence (left) and presence of DAG (5 wt%)/mini-MPIase-3 (5 wt%) (right) at 30 °C. Scale bar = 50 nm. (b) The average vesicle size of EPL liposome in the absence and presence of DAG and/or mini-MPIase-3 (Fig. S3b) determined by DLS. The error bars show the standard deviation of three experiments.

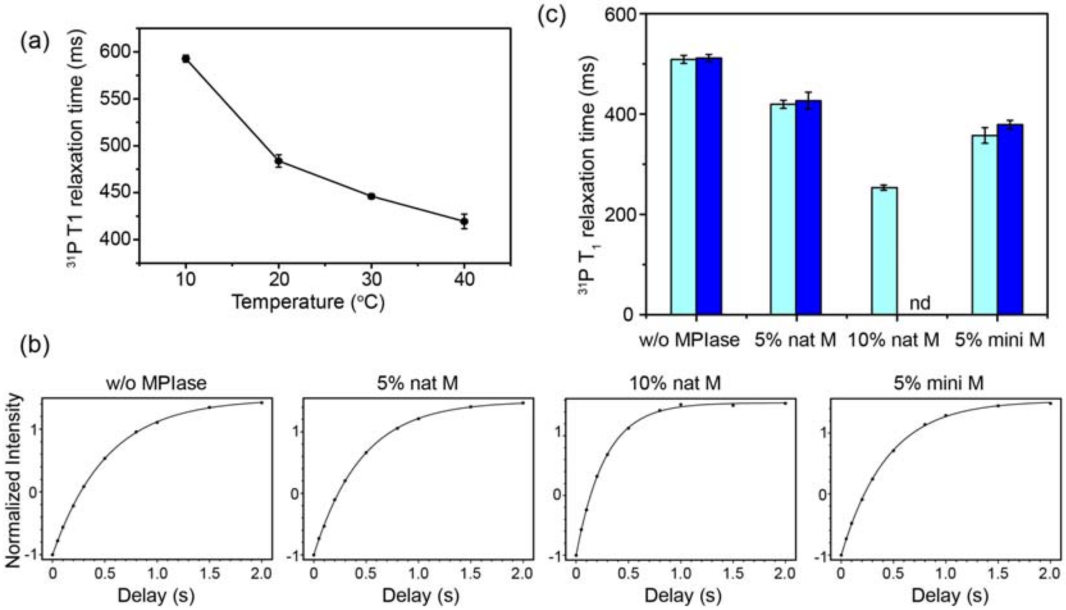

Figure S4. (a) ^{31}P T_1 relaxation times of EPL liposomes in the presence of natural MPIase (5 wt%) as a function of temperature. Decrease of T_1 with increasing temperature indicates that the longer T_1 means slower motion in this sample. (b) Representative normalized signal intensity curves in the inversion recovery experiment of EPL liposomes in the absence and presence of natural MPIase (5 wt%), natural MPIase (10 wt%), and mini-MPIase-3 (5 wt%) at 30 °C. (c) Effects of DAG, mini-MPIase-3 and natural MPIase on the ^{31}P T_1 values of EPL liposomes at 40 °C. From the left, the results of ^{31}P T_1 values in the following conditions are shown in the presence (light blue) and absence (blue) of DAG: without MPIase, natural MPIase (5 wt%), natural MPIase (10 wt%), and mini-MPIase-3 (5 wt%).

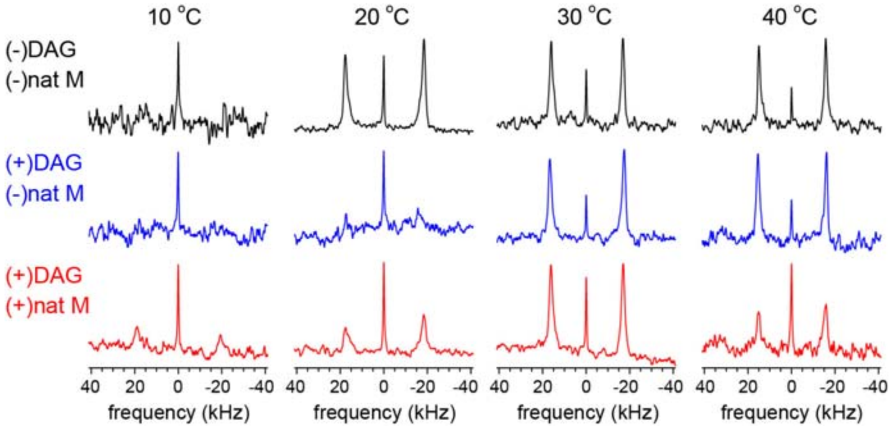

Figure S5. The quadrupole splitting of ^{2}H spectra at the designated temperature in the absence (black) and presence of DAG (5 wt%) (blue), and DAG (5 wt%)/natural MPIase (5 wt%) (red).

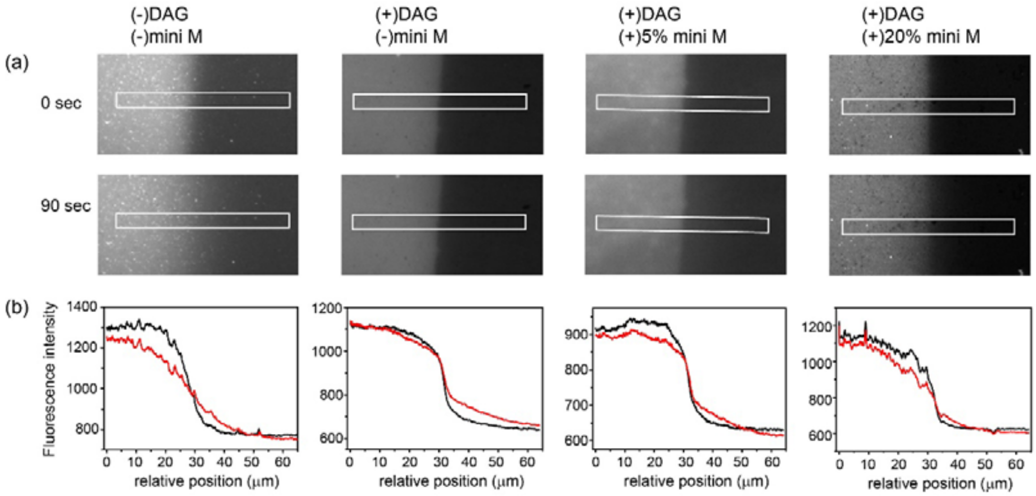

Figure S6. FRAP images for TR-DHPE in EPL SPBs in the absence and presence of DAG (5 wt%), DAG (5 wt%)/mini-MPIase-3 (5 wt%), or DAG (5 wt%)/mini-MPIase-3 (20 wt%) at 30 °C. The left half of the images was bleached, and images were taken immediately after bleaching (0 sec after bleaching) and 90 sec after bleaching. (b) Fluorescence intensity profile for a 65 μm stripe in *x* across the bleached region as shown in (a) at 0 (black) and 90 (red) sec after bleaching.

REFERENCES

S1. Nomura, K., G. Corzo, T. Nakajima, and T. Iwashita. 2004. Orientation and pore-forming mechanism of a scorpion pore-forming peptide bound to magnetically oriented lipid bilayers. Biophys. J. 87:2497-2507.

S2. Nomura, K., G. Ferrat, T. Nakajima, H. Darbon, T. Iwashita, and G. Corzo. 2005. Induction of morphological changes in model lipid membranes and the mechanism of membrane disruption by a large scorpion-derived pore-forming peptide. Biophys. J. 89:4067-4080.

S3. Nomura, K., T. Inaba, K. Morigaki, K. Brandenburg, U. Seydel, and S. Kusumoto. 2008. Interaction of lipopolysaccharide and phospholipid in mixed membranes: solid-state 31P-NMR spectroscopic and microscopic investigations. Biophys. J. 95:1226-1238.

S4. Fung, B. M., A. F. Khitrin, and K. Ermolaev. 2000. An improved broadband decoupling sequence for liquid crystals and solids. J. Magn. Reson. 142:97-101.

S5. Morcombe, C. R., and K. W. Zilm. 2003. Chemical shift referencing in MAS solid state NMR. J. Magn. Reson. 162:479-486.

S6. Seelig, J. 1977. Deuterium magnetic resonance: theory and application to lipid membranes. Q. Rev. Biophys. 10:353-418.

S7. Vold, R. L., J. S. Waugh, M. P. Klein, and D. E. Phelps. 1968. Measurement of Spin Relaxation in Complex Systems. J. Chem. Phys. 48:3831.

S8. Nomura, K., Y. Tanimoto, F. Hayashi, E. Harada, X. Y. Shan, M. Shionyu, A. Hijikata, T. Shirai, K. Morigaki, and K. Shimamoto. 2017. The Role of the Prod1 Membrane Anchor in Newt Limb Regeneration. Angew. Chem. Int. Ed. Engl. 56:270-274.

S9. Shimanouchi, T., H. Kawasaki, M. Fuse, H. Umakoshi, and R. Kuboi. 2013. Membrane fusion mediated by phospholipase C under endosomal pH conditions. Colloids and surfaces. B, Biointerfaces 103:75-83.

S10. Parasassi, T., G. De Stasio, A. d'Ubaldo, and E. Gratton. 1990. Phase fluctuation in phospholipid membranes revealed by Laurdan fluorescence. Biophys. J. 57:1179-1186.

S11. Bai, J., and R. E. Pagano. 1997. Measurement of spontaneous transfer and transbilayer movement of BODIPY-labeled lipids in lipid vesicles. Biochemistry 36:8840-8848.

S12. Nakao, H., K. Ikeda, Y. Ishihama, and M. Nakano. 2016. Membrane-Spanning Sequences in Endoplasmic Reticulum Proteins Promote Phospholipid Flip-Flop.

Biophys. J. 110:2689-2697.

S13. Ueda, Y., A. Makino, K. Murase-Tamada, S. Sakai, T. Inaba, F. Hullin-Matsuda, and T. Kobayashi. 2017. Sphingomyelin regulates the transbilayer movement of diacylglycerol in the plasma membrane of Madin-Darby canine kidney cells. FASEB J. 27:3284-3297.

S14. Merzlyakov, M., E. Li, and K. Hristova. 2006. Directed assembly of surface-supported bilayers with transmembrane helices. Langmuir 22:1247-1253.

S15. Okazaki, T., T. Inaba, Y. Tatsu, R. Tero, T. Urisu, and K. Morigaki. 2009. Polymerized lipid bilayers on a solid substrate: morphologies and obstruction of lateral diffusion. Langmuir 25:345-351.

S16. website of Avanti Polar Lpids : https://avantilipids.com/.
