# Supplementary material for: Alteration of Membrane Physicochemical Properties by Two Factors for Membrane Protein Integration
Source: Biophys J. 2019 May 21;117(1):99–110. doi: 10.1016/j.bpj.2019.05.014 (PMC6626835; doi:10.1016/j.bpj.2019.05.014)
Supplement: Document S2. Article plus Supporting Material [file mmc2.pdf]

Biophysical Journal
Article

Biophysical Society

Alteration of Membrane Physicochemical Properties by Two Factors for Membrane Protein Integration

Kaoru Nomura,[1,*] Toshiyuki Yamaguchi,[1] Shoko Mori,[1] Kohki Fujikawa,[1] Ken-ichi Nishiyama,[2] Toshinori Shimanouchi,[3] Yasushi Tanimoto,[4] Kenichi Morigaki,[5] and Keiko Shimamoto[1,*]
[1]Bioorganic Research Institute, Suntory Foundation for Life Sciences, Kyoto, Japan; [2]Department of Biological Chemistry and Food Sciences, Faculty of Agriculture, Iwate University, Morioka, Iwate, Japan; [3]Graduate School of Environmental Science, Okayama University, Okayama, Japan; [4]Graduate School of Agricultural Science and [5]Biosignal Research Center, Kobe University, Kobe, Japan

ABSTRACT After a nascent chain of a membrane protein emerges from the ribosomal tunnel, the protein is integrated into the cell membrane. This process is controlled by a series of proteinaceous molecular devices, such as signal recognition particles and Sec translocons. In addition to these proteins, we discovered two endogenous components regulating membrane protein integration in the inner membrane of *Escherichia coli*. The integration is blocked by diacylglycerol (DAG), whereas the blocking is relieved by a glycolipid named membrane protein integrase (MPIase). Here, we investigated the influence of these integration-blocking and integration-promoting factors on the physicochemical properties of membrane lipids via solid-state NMR and fluorescence measurements. These factors did not have destructive effects on membrane morphology because the membrane maintained its lamellar structure and did not fuse in the presence of DAG and/or MPIase at their effective concentrations. We next focused on membrane flexibility. DAG did not affect the mobility of the membrane surface, whereas the sugar chain in MPIase was highly mobile and enhanced the flexibility of membrane lipid headgroups. Comparison with a synthetic MPIase analog revealed the effects of the long sugar chain on membrane properties. The acyl chain order inside the membrane was increased by DAG, whereas the increase was cancelled by the addition of MPIase. MPIase also loosened the membrane lipid packing. Focusing on the transbilayer movement, MPIase reduced the rapid flip-flop motion of DAG. On the other hand, MPIase could not compensate for the diminished lateral diffusion by DAG. These results suggest that by manipulating the membrane lipids dynamics, DAG inhibits the protein from contacting the inner membrane, whereas the flexible long sugar chain of MPIase increases the opportunity for interaction between the membrane and the protein, leading to membrane integration of the newly formed protein.

INTRODUCTION

It is important for membrane proteins to be correctly integrated into biomembranes for their proper functioning. The fundamental mechanisms for membrane integration of membrane proteins are thought to be conserved from prokaryotes to eukaryotes because all organisms use homologous complexes of channel proteins termed translocons. In the inner membrane of *Escherichia coli*, many membrane proteins are integrated into the membrane with the help of signal recognition particle (SRP), its receptor, and the Sec translocon complex (Sec/SRP-dependent membrane integration pathway) (1–3) (Fig. 1 *a*). On the other hand, some small membrane proteins containing one or two transmembrane domain(s) followed by a short C-terminus do not require their help (Sec/SRP-independent membrane integration pathway) (4–7) (Fig. 1 *a*). In the Sec/SRP-independent process, it was previously thought that these membrane proteins spontaneously integrate into the membrane. For example, 3L-Pf3 coat protein, which is a mutant version of bacteriophage Pf3 coat protein and has been used as a model substrate, was reported to integrate spontaneously into liposomes composed only of a commercially available mixture of polar lipids extracted from *E. coli* (EPL) (https://avantilipids.com/). However, it became evident that the Sec/SRP-dependent and -independent membrane integration into EPL liposomes cannot be reproduced in a precise manner. For example, even Sec/SRP-dependent proteins such as mannitol permease spontaneously integrated into membranes in the absence of SRP receptor, SRP, or SecYEG. Subsequently, diacylglycerol (DAG) was identified as the factor that blocks such unregulated integration in the *E. coli* inner membrane (8,9). The addition of a physiological concentration of DAG (2–3 weight percentage (wt%) of EPL) into EPL liposomes completely inhibited

Submitted December 13, 2018, and accepted for publication May 14, 2019.
*Correspondence: nomura@sunbor.or.jp or shimamot@sunbor.or.jp
Editor: Timothy Cross.
https://doi.org/10.1016/j.bpj.2019.05.014
© 2019 Biophysical Society.
This is an open access article under the CC BY license (http://creativecommons.org/licenses/by/4.0/).

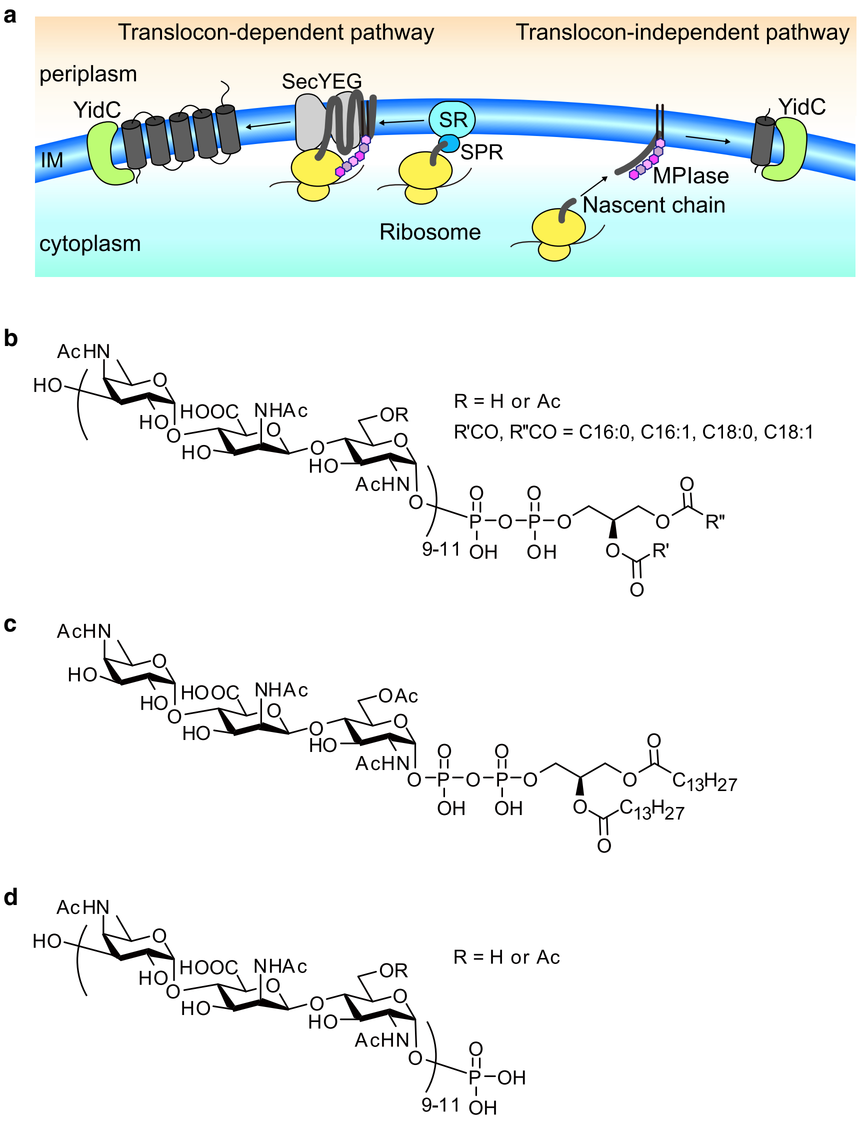

FIGURE 1 (*a*) Schematic representation of the two major membrane protein integration pathways in *E. coli.* In the translocon-dependent pathway, a signal peptide appearing from the ribosome is recognized by the signal recognition particle (SRP), and then the ribosome-nascent chain (RNC)-SRP complex docks to the SRP receptor (SR). RNC is further transferred to the SecYEG translocon and then positioned in the inner membrane (IM) by an insertase, YidC. In the translocon-independent pathway, the nascent protein is released from the ribosome and can integrate into the membrane without SRP-SR and SecYEG complexes. In both pathways, membrane protein integrase (MPIase) is indispensable. (*b–d*) Shown are the molecular structures of natural MPIase (*b*) and its analogs, mini-MPIase-3 (*c*) and Polysac-P (*d*). In natural MPIase and Polysac-P (*b* and *d*), about one-third of the six position on GlcNac residues were estimated to be *O*-acetylated. The number of repeating trisaccharide units ranges from 7 to 14 but most are 9–11. Polysac-P was obtained by pyrophosphatase digestion of natural MPIase (12,13).

the unregulated integration of both Sec/SRP-dependent and -independent proteins. It is well known that DAG plays important roles in lipid synthesis and serves as a second messenger to activate protein kinase C (10). However, the mechanism of the protein membrane integration blockage by DAG has yet to be resolved.

Moreover, we found that protein integration into the DAG-containing EPL liposomes was restored by the addition of *E. coli* inner membrane extracts (11,12). Because this indicates that the integration-promoting factor, termed “membrane protein integrase,” is contained in the *E. coli* inner membrane, we purified the responsible component from extracts. Consequently, we revealed that the integrase, named “MPIase” after its function, does not contain a peptide component. Surprisingly, it is a glycolipid composed of a DAG anchor moiety, a sugar chain consisting of around 10 trisaccharide units, and a pyrophosphate linker (Fig. 1 *b*) (12). The DAG anchor moiety contains fatty acids such as C16:0, C16:1, C18:0, and C18:1, which are typical *E. coli* membrane lipid components. From structure-activity relationship studies, we deduced that the sugar chain of membrane protein integrase (MPIase) binds to the membrane protein and inhibits protein aggregation, similar to a chaperone (13). By adding both DAG and MPIase into EPL liposomes, we successfully obtained reconstituted systems for Sec/SRP-dependent and -independent membrane integration. When we added natural MPIase purified from *E. coli* inner membranes into DAG-containing EPL liposomes, the membrane integration of 3L-Pf3 coat protein increased dose dependently (11). In addition, we recently synthesized a minimal unit of MPIase called mini-MPIase-3 (Fig. 1 *c*), which possesses only one trisaccharide unit and a pyrophospholipid moiety (13). The integration by mini-MPIase-3 reached almost comparable levels to that by natural MPIase, although a higher dose was required (Fig. S1). The anchorless analog of MPIase (Polysac-P) (Fig. 1 *d*), which

corresponds to the monophosphorylated sugar chain moiety of natural MPIase, did not show membrane integration activity, although it inhibited aggregation of the substrate protein. These results indicated that the anchoring of MPIase in the membrane by a lipid moiety is essential for membrane protein integration and that mini-MPIase-3 anchored in the membrane has similar effects on the membrane to natural MPIase.

We speculated that alteration of the membrane physicochemical properties by DAG or MPIase is relevant to membrane protein integration. Therefore, it is possible that by comparing the influence of DAG and natural MPIase/mini-MPIase-3 on the physicochemical properties of liposomes, we could clarify the molecular mechanisms of the blockade and the promotion of membrane protein integration. DAG has a simple molecular structure consisting of two fatty acid chains covalently bonded to a glycerol moiety through ester bonds. Its small polar headgroup without a phosphate group differs from the structure of other membrane lipids. Owing to its unique molecular structure, DAG is known to increase acyl chain order, reduce lateral diffusion of membrane lipids, and have a rapid flip-flop motion (14,15). In contrast, because of the additional long sugar chain and pyrophosphate, MPIase should show different effects from DAG on membranes, despite the fact that MPIase also contains the DAG substructure in its molecule.

In this study, we focused on several membrane properties, such as membrane morphology, the flexibility of the sugar chain, the mobility of the headgroups of phospholipids, the ordering of the phospholipid acyl chain, membrane packing, the flip-flop rate of DAG, and lateral diffusion of membrane lipids. Solid-state NMR and fluorescence studies are a valid approach to this aim. We revealed that DAG and MPIase showed the opposite effects on the physicochemical properties of membranes, suggesting a mutual correlation to the blockage and recovery of the membrane integration of proteins. So far, studies of protein membrane integration have been mainly performed from the viewpoint of the behavior of substrate proteins (for example, quantification of integrated proteins and the identification of the regions of protein insertion in the membrane) (3–12). In addition, the static/dynamic structure of relevant proteins, such as SRP, Sec translocon (with or without substrates), ribosomes, and their complexes (16–23) has been investigated. To the best of our knowledge, this is the first study to elucidate the protein membrane integration mechanism from the viewpoint of membrane physicochemical properties.

MATERIALS AND METHODS

Materials

Natural MPIase was purified from MC4100 as described (11). To obtain a fully ^{13}C-labeled MPIase, MC4100 was propagated in cell growth media containing uniformly ^{13}C-labeled glucose. MPIase analogs were synthesized as described (13). EPL, 1,2-dimyristoyl-*sn*-glycero-3-phosphocholine, 1,2-dimyristoyl-*sn*-glycerol, and 1-palmitoyl-2-{12-[(7-nitro-2-1,3-benzoxadiazol-4-yl)amino]dodecanoyl}-*sn*-glycero-3-phosphocholine (C_{12}-NBD-PC) were purchased from Avanti Polar Lipids (Alabaster, AL). EPL consists of ∼67% of phosphatidylethanolamine, 23.2% of phosphatidylglycerol, and 9.8% of cardiolipin (https://avantilipids.com/). The average fatty acid composition of each natural phospholipid is shown in Table S1. 1,2-Dimyristoyl-*sn*-glycero-3-phosphoethanolamine (DMPE) was purchased from Olbracht Serdary Research Laboratories (Toronto, Canada), and all lipids were used without further purification. Selectively deuterated DMPE (4-d_2-DMPE) was synthesized as described (24). Laurdan was purchased from Chemodex (Hamburg, Germany). 1-Palmitoyl-2-{12-[(7-nitro-2-1,3-benzoxadiazol-4-yl)amino]dodecanoyl}-*sn*-glycerol (C_{12}-NBD-DAG) was prepared from C_{12}-NBD-PC using phospholipase C digestion. Phospholipase C from *Clostridium perfringens* and deuterium-depleted water were purchased from Sigma-Aldrich (St. Louis, MO). Sodium dithionite (sodium hydrosulfite) was purchased from Tokyo Chemical Industry (Tokyo, Japan). Sodium dodecyl sulfate was purchased from Nacalai Tesque (Kyoto, Japan). Texas Red 1,2-dihexadecanoyl-*sn*-glycero-3-phosphoethanolamine (excitation/emission: 583 nm/601 nm) was purchased from Molecular Probes (Eugene, OR). Hellmanex solution was purchased from Hellma (Müllheim, Germany). 1,2-Dimyristoyl-*sn*-glycerol was used as a representative of DAG (and is referred to as DAG throughout this study, unless otherwise designated) because DAGs possessing C8–C18 fatty acids showed almost the same blocking activities for membrane protein integration (8). The amount of the compound in the membrane was expressed as wt% to EPL.

Expanded methods are provided in the Supporting Methods.

RESULTS

Membrane morphology

To clarify the effect of two membrane protein integration factors, DAG and MPIase, on the membrane, we first investigated the membrane morphology of liposomes prepared from EPL in the absence and presence of DAG and/or natural MPIase. The ^{31}P NMR spectrum of EPL liposomes under static conditions showed a magnetically aligned, axially symmetric lamellar pattern (Fig. 2 *a*, *top*). Multilamellar vesicles composed of EPL tend to align and be prolate in the magnetic field (25). Even in the presence of DAG (5 wt%) and/or natural MPIase (5 wt%), the lamellar pattern was retained (Fig. 2 *a*). These results demonstrated that the EPL membrane phase was not altered significantly by the addition of these two factors at 5 wt%. Spectra for higher concentrations are shown in Fig. S2. Increasing the DAG concentration to 20 wt% destroyed the lamellar structure, whereas even 50 wt% of mini-MPIase-3 did not alter the membrane phase. Dynamic light scattering measurement and the observation of cryo-transmission electron microscope images showed that EPL liposomes in each sample formed single large unilamellar vesicles (LUVs), regardless of the presence of DAG and/or mini-MPIase-3, and no significant difference in the average size of liposomes was observed between each sample (Fig. S3). DAG is known to destabilize the lipid bilayer structure and lead to membrane fusion (26–28). Therefore, we confirmed whether DAG causes membrane fusion by analyzing the percentage of membrane fusion by the cobalt (II)/calcein method

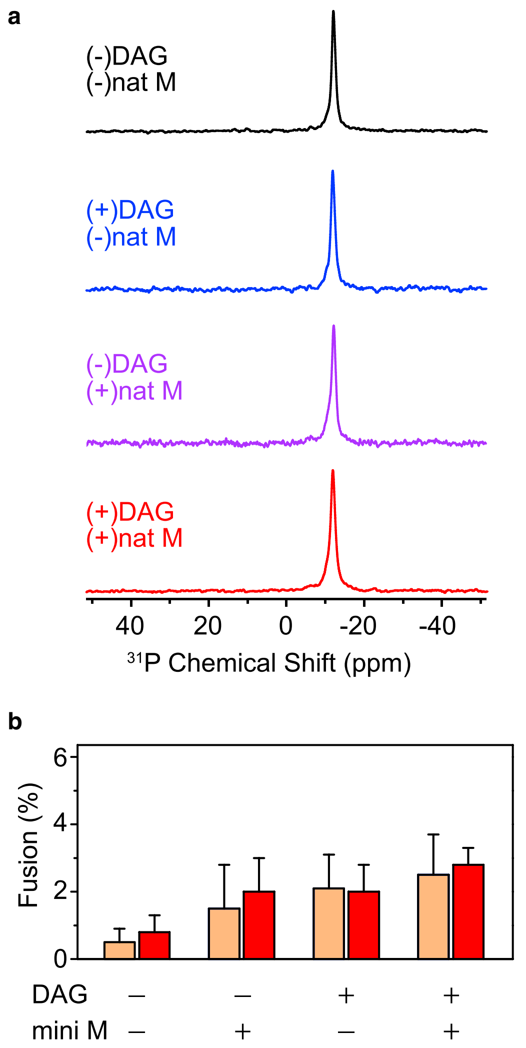

FIGURE 2 (*a*) ^{31}P NMR spectra of EPL liposomes in the absence (*black*) and presence of DAG (5 wt%) (*blue*), natural MPIase (5 wt%) (*purple*), and DAG (5 wt%)/natural MPIase (5 wt%) (*red*) at 30°C. (*b*) The percentage of the fusion of EPL liposomes in the absence and presence of DAG (5 wt%) and/or mini-MPIase-3 (5 wt%) was determined using the cobalt/calcein method at 30 (*light salmon*) or 37°C (*red*).

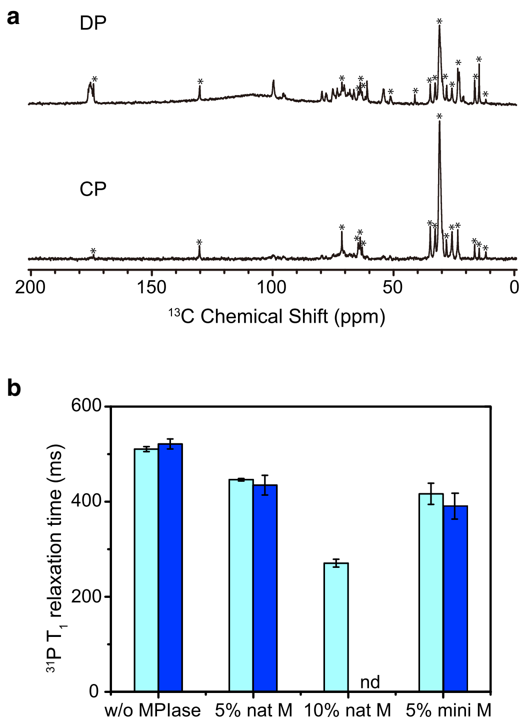

FIGURE 3 (*a*) Direct polarization (DP) (*top*) and cross-polarization (CP) (*bottom*) ^{13}C NMR spectra of EPL liposomes in the presence of uniformly ^{13}C-labeled natural MPIase (5 wt%) under magic angle spinning with a spinning speed of 5 kHz at 30°C. The peaks from EPL are marked as with an asterisk. (*b*) The effect of mini-MPIase-3, natural MPIase, and DAG on the ^{31}P T_1 values of EPL liposomes at 30°C is shown. From the left, results of ^{31}P T_1 values in the following samples are shown in the presence (*light blue*) and absence (*blue*) of DAG: without MPIase, natural MPIase (5 wt%), natural MPIase (10 wt%), and mini-MPIase-3 (5 wt%). The error bars show the SD of at least three experiments. Compared to the T_1 values at 40°C (Fig. S4 *b*), T_1 values at 30°C for all samples showed higher values, in which higher T_1 values indicate slower motion for all samples.

(Fig. 2 *b*). The percentage of LUV membrane fusion was lower than 3%, regardless of the presence or absence of DAG and/or MPIase. We concluded that neither DAG nor MPIase caused membrane morphological changes or membrane fusion at physiological concentrations.

^{13}C NMR spectra of MPIase in liposomes

To understand the relative molecular mobility of MPIase and membrane lipids, we measured the ^{13}C cross-polarization (CP) and direct polarization (DP) NMR spectra of EPL liposomes containing uniformly ^{13}C-labeled natural MPIase (5 wt%) under magic angle spinning at 30°C (Fig. 3 *a*). It is generally known that NMR signals of all components are observed using DP, whereas only those of rigid components are observed using CP. In Fig. 3 *a*, signals of membrane lipids were observed both in the DP (top) and CP (bottom) spectra, confirming that lipids in the liposomes are rigid. On the other hand, signals originating from the sugar chain disappeared in the CP spectrum, whereas they were present in the DP spectrum. This indicates that the sugar chain is highly mobile and does not interact with the membrane surface. The observed ^{13}C NMR signals undoubtedly originated from the membrane-bound MPIase as we could not detect any signals in the supernatant after ultracentrifugation of the suspension of multilamellar vesicles.

^{31}P T_1 relaxation times of liposomes

To analyze the dynamics of phospholipid headgroups in membranes, we measured ^{31}P T_1 relaxation times (Fig. 3 *b*). The ^{31}P T_1 relaxation times in the EPL/natural MPIase (5 wt%) liposomes decreased with the increasing temperature (Fig. S4 *a*). Thus, the longer T_1 means a slower motion for the ^{31}P nucleus in the headgroup in our measurements

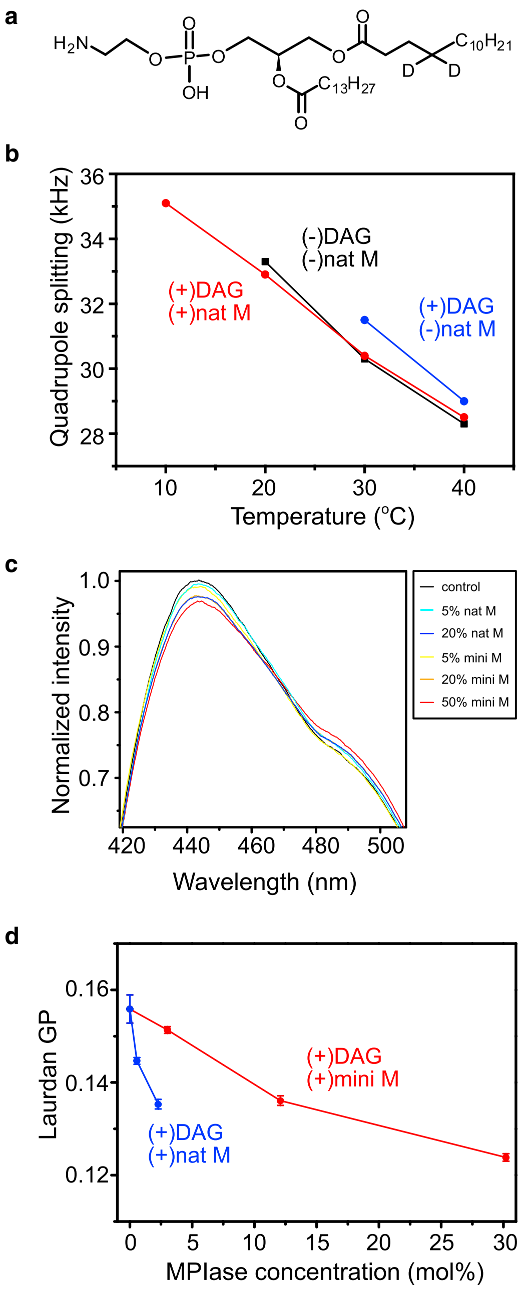

FIGURE 4 (*a*) Molecular structure of selectively ^{2}H-labeled DMPE (4-d_2-DMPE) used in ^{2}H NMR measurements. (*b*) Shown is the temperature dependence of the quadrupole splitting values in the ^{2}H static spectra of EPL/^{2}H-labeled DMPE/1,2-dimyristoyl-*sn*-glycero-3-phosphocholine (93.3:5:1.7 w/w/w) liposomes in the absence (*black*) and presence of DAG (5 wt%) (*blue*) and DAG (5 wt%)/natural MPIase (5 wt%) (*red*). As shown in Fig. S5, values for the control sample at 10°C and those for the DAG-containing sample at 10 and 20°C could not be measured by the broadening of peaks. (*c*) Shown are the emission spectra of laurdan in EPL/DAG (100:5 w/w), in the absence (*black*) and presence of 5 wt% (*light blue*), 20 wt% (*blue*) of natural MPIase, and 5 wt% (*yellow*), 20 wt% (*orange*), and 50 wt% (*red*) of mini-MPIase-3 at 30°C. Fluorescence intensity values were normalized by the peak area from 400 to 600 nm for each spectrum. Emission spectra were measured at a 360 nm excitation wavelength in all measurements. Data are presented as the means of three independent experiments. (*d*) Shown is the MPIase molar concentration dependence of laurdan GP values, $GP = (I_{440} - I_{490})/(I_{440} + I_{490})$, for natural MPIase (*blue*) and mini-MPIase-3 (*red*) in the presence of 5 wt % DAG calculated from Fig. 4 *c*. The error bars show the SD of three experiments.

(29). We measured the ^{31}P T_1 relaxation times of the EPL membranes in the absence and presence of natural MPIase (5 and 10 wt%) at 30°C (Figs. S4 *b* and 3 *b*). The ^{31}P NMR signal originated from all components in the vesicles; thus, the change in the ^{31}P T_1 relaxation time reflected the bulk properties of the membranes. The T_1 values decreased depending on the amount of natural MPIase. This indicates that MPIase increases the headgroup mobility of the membrane lipids. The T_1 values were not significantly changed by the addition of DAG, indicating that DAG did not affect the headgroup mobility of lipids. The decrease in the T_1 value by mini-MPIase-3 (5 wt%) was almost comparable to that by natural MPIase (5 wt%) (Fig. 3 *b*).

Membrane lipid acyl chain ordering

We next investigated the influence of DAG and MPIase on the ordering of membrane lipid acyl chains. It is known that the value of quadrupolar splitting in the static ^{2}H spectra increases according to the ordering of acyl chains (30). Thus, we measured the ^{2}H NMR spectra of EPL liposomes containing ^{2}H-labeled DMPE (Fig. 4 *a*). Because the ^{2}H spectrum of the control liposomes at 10°C and those for the DAG-containing liposomes at 10 and 20°C were broadened because of the decrease of acyl chain motion (Fig. S5), accurate values of quadrupole splitting could not be obtained. The value for the control EPL liposomes decreased with the increasing temperature, whereas the value increased after adding 5 wt% DAG at every temperature (Fig. 4 *b*, *blue*). This suggested the ordering of membrane lipids in the presence of DAG. Moreover, after the addition of natural MPIase (5 wt%) into the DAG-containing liposomes, the quadrupole splitting values (Fig. 4 *b*, *red*) returned to the original values (Fig. 4 *b*, *black*), suggesting that MPIase compensated for the increase in membrane ordering by DAG.

Packing of membrane lipids

The acyl chain ordering of phospholipids likely alters membrane lipid packing. Thus, we measured the emission spectra of laurdan in EPL liposomes in the presence of DAG and MPIase. Laurdan is a widely used probe for assessing membrane lipid packing because its fluorescence spectra changes in response to the water content of its surroundings (31–34). Further, laurdan probes around the hydrophilic-hydrophobic interface of lipid bilayers (35,36). Fig. 4 *c* shows the emission spectra of laurdan in the 5 wt % DAG-containing EPL liposomes in the absence (*black*) and presence of 5 wt% (*light blue*) or 20 wt% (*blue*) natural MPIase at 30°C. The dose-dependent red shift of laurdan's

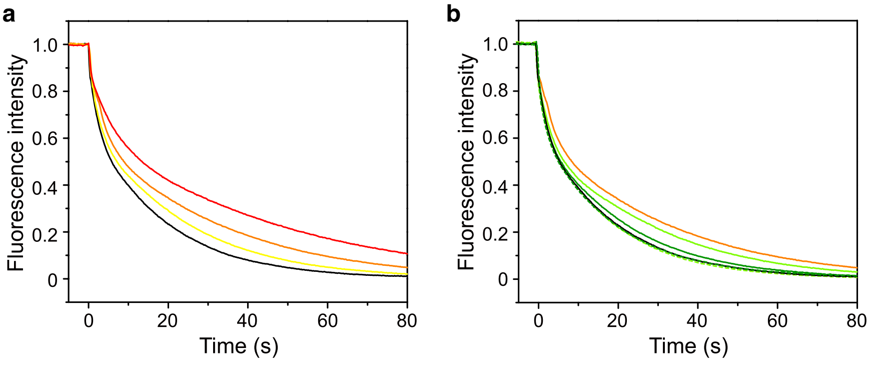

FIGURE 5 Normalized fluorescence decay curve of C_{12}-NBD-DAG in EPL/C_{12}-NBD-DAG (100:1 w/w) liposomes (*a*) in the presence of various mini-MPIase-3 concentrations of 0 (*black*), 5 wt% (*yellow*), 20 wt% (*orange*), and 50 wt% (*red*) at 150 mM NaCl and (*b*) in the presence of mini-MPIase-3 (20 wt%) at various NaCl concentrations of 150 mM (*solid orange*), 300 mM (*solid light green*), and 500 mM (*solid green*) and in the absence of mini-MPIase-3 at various NaCl concentrations of 150 mM (*solid black*), 300 mM (*broken light green*), and 500 mM (*broken green*). All spectra were acquired at 30°C at excitation and emission wavelengths of 475 and 530 nm, respectively. Fluorescence intensities were normalized to the value right before injecting the dithionite after correcting the equilibrium values to zero. Data are presented as the means of three independent experiments.

emission spectrum by natural MPIase indicates that natural MPIase loosened the lipid packing around the interfacial region inside the membrane (Fig. 4 *c*). Mini-MPIase-3 in the DAG-containing EPL liposomes also dose dependently red shifted the emission spectra (Fig. 4 *c*), and the effect of mini-MPIase-3 was comparable to that of natural MPIase at the same wt%. The generalized polarization (GP) values of laurdan [$GP = (I_{440} - I_{490})/(I_{440} + I_{490})$] quantify shifts in the emission spectrum of laurdan. When we compared the GP values calculated from Fig. 4 *c* based on the number of molecules involved in the membrane, natural MPIase was more potent than mini-MPIase-3 (Fig. 4 *d*).

Inhibition of DAG flip-flop motion by MPIase

DAG is known to have a rapid flip-flop rate in membranes because of its compact shape (37,38). The rapid flip-flop motion of DAG may affect protein membrane integration. Therefore, we examined how MPIase affects the DAG flip-flop rate (37,38). Fig. 5 *a* shows the normalized fluorescence decay curve of C_{12}-NBD-DAG in EPL liposomes by dithionite. The time constants of the flip-flop movement, t_{flop}, and dithionite quenching of C_{12}-NBD-DAG in the outer leaflet, t_q, were obtained by curve fitting using Eq. S4. Both t_q and t_{flop} values increased with increasing mini-MPIase-3 concentration (Table S2). To evaluate the inhibition of dithionite quenching by mini-MPIase-3, the t_q value of C_{12}-NBD-PC-containing liposomes was measured because C_{12}-NBD-PC does not undergo flip-flop movement at this timescale, and t_{flop} can be ignored. The t_q value of C_{12}-NBD-PC-containing liposomes was also increased by the addition of mini-MPIase-3, suggesting that the steric and/or ionic repulsion by sugar chains interfered with dithionite quenching. The t_{flop} values of C_{12}-NBD-DAG-containing liposomes, which increased depending on the mini-MPIase-3 concentration, could also be affected by the delay in quenching. Nevertheless, we concluded that the flip-flop motion of DAG was reduced by mini-MPIase-3 because the increment of t_{flop} values was significantly larger than that of t_q values (Table S2). We further measured the quenching of C_{12}-NBD-DAG at various NaCl concentrations (150, 300, 500 mM) in the absence and presence of 20 wt% of mini-MPIase-3 (Fig. 5 *b*). The t_{flop} values in the absence of mini-MPIase-3 were not affected by the NaCl concentration (Table S3). In contrast, t_{flop} values in the presence of 20 wt% of mini-MPIase-3 gradually decreased as the concentration of NaCl increased and recovered to the value in the absence of mini-MPIase-3.

Effect of DAG and MPIase incorporation on lateral diffusion of lipids

We speculated that the heightened packing of the membrane lipids by DAG and its inhibition by mini-MPIase-3 may influence the lateral diffusion rate of membrane lipids. Therefore, we analyzed the effects of DAG and mini-MPIase-3 on the lateral diffusion of lipids using the boundary profile evolution method in supported planar lipid bilayers (SPBs) (Fig. S6). Fig. 6 shows the lipid lateral diffusion coefficient, *D*, of EPL SPBs in the absence and presence of DAG

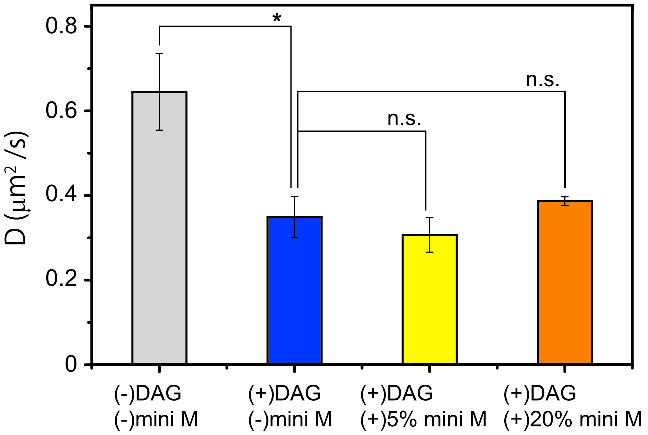

FIGURE 6 The lipid lateral diffusion coefficient *D* of EPL supported planar lipid bilayers (SPBs) in the absence (*gray*) and presence of DAG (5 wt%) (*blue*), DAG (5 wt%)/mini-MPIase-3 (5 wt%) (*yellow*), or DAG (5 wt%)/mini-MPIase-3 (20 wt%) (*orange*) at 30°C. The error bars show the SE of at least three samples and five spot variations. $*p < 0.05$; *t*-test.

(5 wt%) and mini-MPIase-3 (5 or 20 wt%). Without additives, the lateral diffusion coefficient was 0.65 $\mu m^2/s$, which is the typical value of EPL (39,40). In the presence of 5 wt% DAG, the diffusion coefficient significantly decreased to 0.35 $\mu m^2/s$, indicating that DAG disturbed the lateral diffusion. When we further added mini-MPIase-3 (5 or 20 wt%) into the DAG-containing EPL membranes, the diffusion coefficients were 0.31 and 0.39 $\mu m^2/s$, respectively. Therefore, unlike DAG, the influence of mini-MPIase-3 on the rate of lateral diffusion was not clear.

DISCUSSION

Sec/SRP-independent integration of membrane proteins into biomembranes (Fig. 1 *a*) is an essential process in vivo; however, its mechanism has yet to be resolved. The Sec/SRP-independent integration of substrate proteins we have examined to date, such as Pf3 procoat protein, its mutant protein (3L-Pf3), M13 procoat protein, and the F_0 c subunit of F_0F_1 ATPase, was blocked by the addition of physiological concentrations of DAG, whereas the addition of MPIase into the DAG-containing membranes restored the integration in each case (9,11,41). These results suggested that the Sec/SRP-independent integration was generally controlled by DAG and MPIase. Structure-activity relationship studies revealed that the anchoring of MPIase in the membrane by its lipid moiety is essential for the membrane protein integration (13). The *E. coli* cell membrane is composed of a wide variety of lipid molecules that dynamically interact with each other. Two important endogenous molecules, DAG and MPIase, whose headgroup sizes are completely different, are suggested to alter membrane properties, evidenced by reports that some membrane properties, such as membrane morphology, phase transition temperature, and acyl chain order of lipids, are related to lipid head size (42–44). Alteration of the physicochemical properties of membranes induced by these two molecules is highly likely to affect the membrane integration efficiency. In this study, we examined the effects of DAG and MPIase on the membrane in terms of membrane morphology (Fig. 2), headgroup flexibility (Fig. 3 *b*), acyl chain ordering (Fig. 4 *b*), packing (Fig. 4, *c* and *d*), and the lateral diffusion coefficient (Fig. 6) of membrane lipids. We also studied the mobility of MPIase (Fig. 3 *a*) and the flip-flop rate of DAG (Fig. 5). These properties are all mutually related to the blockage and recovery of membrane integration in the presence of DAG and MPIase.

We investigated the membrane properties by using natural MPIase and mini-MPIase-3, a synthetic analog having the minimal functional structure for the integration activity. Equivalent weights of MPIase and mini-MPIase-3 showed similar effects in ^{31}P NMR relaxation measurement (Fig. 3 *b*) and membrane packing analysis (Fig. 4, *c* and *d*), indicating that mini-MPIase-3 is also available for the study of membrane properties. However, the molecular mass of natural MPIase is ~7000 Da, whereas that of mini-MPIase-3 is ~1300 Da. Because greater than 5 times of mini-MPIase-3 molecules was required to produce an effect comparable to natural MPIase (Fig. 4 *d*), natural MPIase is more potent. The reason for this is suggested to be because its sugar chain length is about 10 times longer than that of mini-MPIase-3.

DAG has a cone shape, whereas MPIase has a reverse cone shape. Therefore, high concentrations of these molecules might disturb the membrane structure. However, the membrane morphology did not change in the presence of only DAG (5 wt%), MPIase (5 wt%), or DAG (5 wt%)/natural MPIase (5 wt%) (Fig. 2 *a*). Regarding DAG, Schorn and Marsh reported that the ^{31}P NMR spectrum of a hydrated 1,2-dimyristoyl-*sn*-glycero-3-phosphocholine (DMPC)-DAG mixture at around 30°C showed a lamellar pattern at a molar ratio of DMPC:DAG at 7:3, but at 4:6, it was altered to be a mixture of lamellar and a small amount of a cylindrical symmetry pattern, and then at 2:8, it showed an isotropic pattern with a small amount of a lamellar pattern (15). Using ^{31}P NMR spectroscopy, we also observed that EPL membranes did not form a pure lamellar structure in the presence of 20 and 50 wt% DAG (Fig. S2, *a* and *b*). Therefore, DAG induces a nonlamellar phase at concentrations higher than 20 wt%. DAG also has the tendency to cause membrane fusion, especially in the presence of phosphatidylethanolamine, because of the perturbation of bilayer structures (26–28). However, the result of the fusion assay using the cobalt-calcein method (Fig. 2 *b*) demonstrated that EPL liposomes containing 5 wt% DAG did not cause membrane fusion. According to the previous study (8), a 5 wt% concentration of DAG is sufficient to inhibit protein integration. Because DAG at this concentration did not influence the membrane morphology, as shown in this study, we can rule out the possibility that the structural change of membranes by DAG inhibits membrane protein integration. Moreover, we found that the membrane morphology was not affected by either 5 wt% of natural MPIase or even 50 wt% of mini-MPIase-3 (Fig. S2, *c* and *d*). We also confirmed that mini-MPIase-3 did not cause membrane fusion with and without DAG (Fig. 2 *b*). Because 5 wt% of natural MPIase or mini-MPIase-3 in liposomes is sufficient to recover membrane integration, we concluded that the regulation of protein integration by MPIase does not originate from membrane morphological changes.

We then analyzed the mobility of the membrane surface (Fig. 3). In the ^{13}C NMR spectra of natural MPIase in the EPL liposomes, the sugar chain peaks appeared in the DP spectrum, whereas they disappeared in the CP spectrum (Fig. 3 *a*). Therefore, the MPIase sugar chain was more flexible than the membrane lipids. Nowacka et al. reported that CP signals of the sugar chain in n-octyl β-D-maltoside were observed only when their correlation times were longer than 1 ms by NMR measurement and simulation study (45). The reported order parameters *S* of the

headgroups of some glycolipids containing one or two sugars on their headgroups are smaller (S = 0.35–0.53) than that of lipids (S = 0.65) in the lipid bilayers, indicating that the sugar chain headgroups show greater fluctuation compared to lipids (46–48). Generally, oligosaccharides continuously change their conformation with a higher degree of motional freedom. The longer the sugar chain, the higher the motional freedom and the greater the fluctuation of the chain (49).

To examine the effect of the sugar chain on the membrane headgroup dynamics, we measured the ^{31}P T_1 relaxation time (Fig. 3 *b*). Both natural MPIase and mini-MPIase-3 made the headgroups of membrane lipids in vesicles more flexible. Natural MPIase was more effective than mini-MPIase-3 when the number of molecules in the liposomes was considered (Fig. 3 *b*). These results indicate that the membrane surface flexibility depends on the length of the sugar chain. The fluctuation of the sugar chain observed in the ^{13}C NMR spectra (Fig. 3 *a*) is highly likely to contribute to the loosening of the membrane surface. On the other hand, DAG had little influence on the T_1 value. This is most likely because its small headgroup is unexposed to the membrane surface.

As for the mobility of the inner part of the membrane, the quadrupole splitting of the ^{2}H NMR spectra increased in the presence of DAG (Fig. 4 *b*), demonstrating that DAG promotes the ordering of lipid acyl chains. A similar increase of membrane lipid ordering in the presence of DAG was reported (15,50). Cholesterol is also known to be one of the most effective molecules in the cell membranes to cause lipid ordering. Lipid ordering can be explained by the "Umbrella model" (51), in which cholesterol is located under the polar headgroups of phospholipids and is shielded from water molecules. The space under the headgroups is densely filled with cholesterol and acyl chains. The lipid ordering by DAG is most likely to be explained by the same model as cholesterol because of the lack of a polar headgroup. The structure of DAG likely favors its fitting under the polar headgroup of other phospholipids, causing the lipid acyl chains to be more ordered.

By contrast, the addition of natural MPIase disordered the lipid acyl chains (Fig. 4 *b*). Besides, the emission spectra of laurdan showed that both natural MPIase and mini-MPIase-3 loosened the packing of membrane lipids (Fig. 4 *c*). Basically, when the membrane consists of lamellar phase-forming lipids like phosphatidylcholine (PC) or nonlamellar phase-forming lipids like DAG, the membrane loses the balance of the volume between their headgroups and acyl chains. To maintain the curvature of membranes as zero, the lateral pressure in the acyl chains is enhanced, resulting in elevated packing stress (52). Therefore, the highly ordered membrane in the presence of DAG, as observed in the ^{2}H NMR measurement in this study, is attributed to packing stress. Furthermore, when we added either natural MPIase or mini-MPIase-3 to the DAG-containing membranes, the large headgroup of the glycolipids would enhance the lateral pressure in the headgroup region. Hence, rebalancing between the headgroup volume and the acyl chain volume in membranes and the release of packing pressure is likely to occur. Consequently, acyl chain disordering and the loosening of membrane packing were observed after adding MPIase to the DAG-containing membranes (Fig. 4).

Dinic et al. reported that the emission spectra of laurdan in LUV were unaltered by the addition of peptides such as mastoparan and bovine prion protein-derived peptide(bPrPp) (33). Antollini et al. also reported that the presence of membrane proteins did not affect the emission spectra of laurdan in liposomes made from tissue lipid extract (31). These results suggested that integrated proteins do not affect membrane lipid packing. On the other hand, N-terminus 18-residue peptide of epsin-1 (EpN18), which is known to promote the direct membrane translocation of cell penetration peptide (CPP), was reported to loosen membrane lipid packing (53). The loosening of membrane packing by EpN18 is assumed to increase the packing defect, which allows the interaction between a peptide main chain with the membrane inner part, facilitating the membrane translocation of CPP. In our study, the flexible sugar chain in MPIase also actively loosened lipid packing around the interfacial region inside the membrane (Fig. 4 *c*). Therefore, MPIase is also likely to allow a substrate protein to approach the membrane interior, promoting membrane integration of the protein.

Finally, we focused on the flip-flop motion and lateral diffusion in the membranes, which would reflect the movement of the whole lipid molecule. It has been reported that the half time of the flip-flop motion of DAG is in the order of seconds to minutes (54,55) and is much faster than that of phospholipids (in the order of days) (56,57). In our study, we demonstrated that mini-MPIase-3 reduced the flip-flop motion of DAG (Fig. 5 *a*). The inhibition of DAG flip-flop motion by MPIase was reversed as the NaCl concentration increased (Fig. 5 *b*). These results indicate that some type of electrostatic interaction, such as intermolecular hydrogen bonds between pyrophosphate or carboxylic acid in MPIase and the hydroxyl group in DAG, might be cleaved under a high salt concentration.

The lateral diffusion of membrane lipids was slowed by DAG incorporation (Fig. 6), which is in agreement with the molecular dynamics simulation by Alwarawrah et al. (14). A decrease in the lateral mobility is likely due to the heightened packing of the hydrophobic segment in the membrane, which is similar to the effect of cholesterol. Both DAG and cholesterol have compact and predominantly hydrophobic molecular structures, which enable them to be buried in the hydrophobic part of the membrane and to move across the bilayer rapidly (58). Recently, it was reported that cholesterol inhibited membrane protein integration like DAG in PC membranes (59). Therefore, it is plausible that

the common effects of DAG and cholesterol on membrane packing function to modulate the protein membrane integration activities. Although it is rather difficult to experimentally correlate the flip-flop motion and lateral mobility, the DAG flip-flop motion might influence the lateral mobility more directly by changing the degree of membrane density fluctuation (60). As soon as a transient space among phospholipids is formed in the membrane, DAG might flip across the lipid bilayer and fill up the space to reduce the unfavorable exposure of the hydrophobic acyl chains. This would attenuate the contact of proteins with the inside of the membrane and block membrane protein integration. In the presence of MPIase, locally unfilled hydrophobic space would be formed in the acyl chain region more frequently by the flexible motion of the MPIase sugar chain, and the unfilled hydrophobic space would be retained longer because of the inhibition of the flip-flop motion of DAG, enabling proteins to integrate more easily into the membrane. In this study, incorporation of mini-MPIase-3 into the EPL/DAG (5 wt%) membrane did not change the lateral diffusion rate significantly (Fig. 6), even though mini-MPIase-3 loosened the lipid packing in the DAG-containing EPL liposomes (Fig. 4 *c*). The lack of rescued lateral mobility by mini-MPIase-3 may be caused by membrane heterogeneity because long distance migration of the probe molecules as measured by fluorescence recovery after photobleaching-boundary profile evolution might be retarded by the presence of obstacles such as nanoscopic or microscopic membrane domains (61,62).

On the basis of our results, we propose the dynamic effects of DAG and MPIase on the integration of small hydrophobic membrane proteins like 3L-Pf3 coat protein, as shown in Fig. 7. In the presence of DAG, the lipid acyl chain becomes ordered (i.e., the membrane is tightly packed). Besides, the rapid flip flop of DAG reduces the lateral diffusion and fills up the unfilled hydrophobic space formed by the exposed acyl chains, resulting in the difficulty of proteins to contact the inside of membranes. On the other hand, when MPIase is present in addition to DAG, the flexible bulky sugar chain of MPIase makes the membrane surface flexible, loosens membrane packing around the interfacial region inside the membrane, disorders membrane lipid acyl chains, and reduces DAG's flip-flop motion. The small hydrophobic membrane proteins prefer to associate with the membrane interior rather than the aqueous environment outside the membrane. Therefore, these proteins tend to integrate into the membrane with the assistance of MPIase. MPIase also maintains the unfilled hydrophobic space by reducing the flip-flop motion of DAG. Thus, the unique molecular structures of DAG and MPIase would be utilized to block and promote membrane protein integration. DAG is a general membrane lipid, whereas MPIase is a specific molecule whose structural features as a glycolipid might be adopted for protein integration.

Further structure-activity relationship studies must be conducted to elucidate the structural requirements that influence the physicochemical properties of membranes. However, it is difficult to use natural MPIase because only a limited amount of natural MPIase can be obtained from the inner membrane of *E. coli*; further, MPIase is a mixture of homologs that are heterogeneous in the number of *O*-Ac groups, glycan length, and types of fatty acids. Compared to natural MPIase, synthetic MPIase analogs can be precisely modified in their functional groups; thus, they would be useful probes in the place of natural MPIase. Therefore, the synthesis of several mini-MPIase-3-analogs is now in progress, in which the pyrophosphate or functional groups in the sugar chain have been modified.

Nishiyama et al. reported that the isolated yield of MPIase was approx. 0.5 wt% of the inner membrane of *E. coli* (11,63). However, the precise amount and its localization

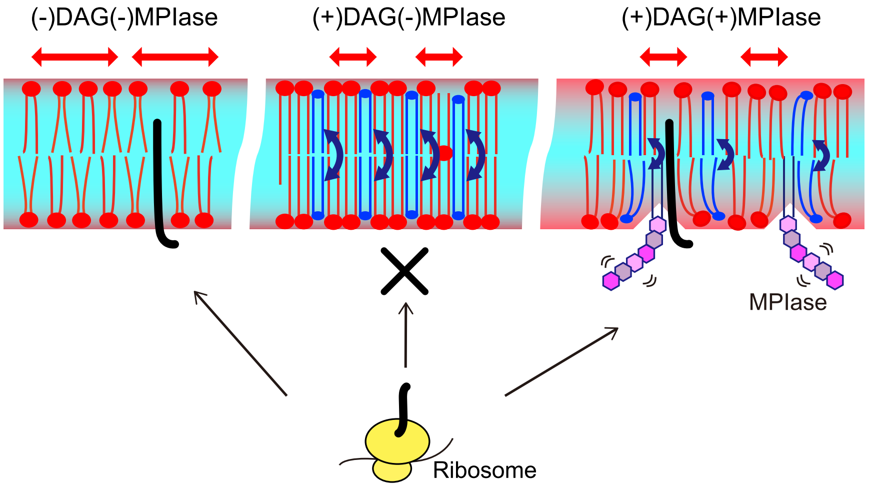

FIGURE 7 Schematic model of the effects of DAG (shown in *blue*) and MPIase (shown in *purple*) on the integration of a small hydrophobic substrate protein (shown in *black*) in the EPL membrane. Blue double-headed arrows show the rate of flip-flop motion of DAG. Red double-headed arrows show the degree of the membrane lipids' lateral diffusion rate. The flexibility of lipids in the membrane is shown as a gradient from light blue to red. Without DAG and MPIase (*left*), the membrane was loosely packed, and lateral diffusion was fast. In the DAG-containing membrane (*middle*), membrane lipids were ordered, DAG's flip-flop motion was very fast, and lateral diffusion was slow. These features would generate difficulty in contacting the nascent proteins with the inside of the membrane. On the other hand, in the presence of MPIase (*right*), the flexible MPIase sugar chain allows phospholipid headgroups to move more flexibly. MPIase also disorders the lipid acyl chain and loosens membrane packing. As a result, MPIase assists the nascent proteins in interacting with the inside of the membrane.

in the inner membrane remain unknown as expression levels vary according to cultivation conditions such as temperature. Although the concentrations in this study (5–20 wt%) may be high compared to the isolated yield, we suggest that the emphasized alterations observed in this study reflect the phenomena in the local region where MPIase functions. We acknowledge the possibility that MPIase assembles on the membrane, and we are conducting experiments to investigate this.

Although we focused on the membrane physicochemical properties, the phenomenon of membrane protein integration cannot be discussed only in the context. We must consider the intermolecular interactions between MPIase and substrates and/or ribosomes. We proposed a mechanism in which a nascent hydrophobic membrane protein synthesized by a ribosome is captured by the long sugar chain of MPIase and is prevented from aggregating by retaining a structure that is capable of integrating into the membrane. The protein would then be attracted by the negative charge of the pyrophosphate group of MPIase and immediately be delivered into the membrane. We are currently examining the molecular interactions between MPIase and substrates in solution to characterize the events preceding the delivery to the membrane.

CONCLUSIONS

The dynamic parameters investigated in this study are important factors in the initial stage of membrane integration. We found that DAG avoids unregulated integration, whereas MPIase restores the favored integration into the membrane by manipulating the dynamics of membrane lipids, as proposed in Fig. 7. We suggest that Sec/SRP-dependent and -independent processes (Fig. 1 *a*) have in common the strategy of controlling membrane dynamics. Additionally, the Sec/SRP-dependent process might require more advantageous interactions between MPIase and Sec, SRP, and YidC proteins on the membrane. At this stage, we have not determined the structure and topology of substrate proteins in the membrane integrated by MPIase, which would also be affected by other membrane components. Solid-state NMR experiments to determine the topology of substrate proteins in the membrane and which residues in the substrate proteins are exposed to the aqueous environment are now in progress. Our results provide the basis for understanding the role of the surrounding environment of substrate proteins and augment the current understanding of the integration mechanism as well as the new biological functions of glycolipids in the context of current studies.

SUPPORTING MATERIAL

Supporting Material can be found online at https://doi.org/10.1016/j.bpj.2019.05.014.

AUTHOR CONTRIBUTIONS

K.N., T.Y., and K.S. designed the research. K.N., T.Y., and S.M. performed and analyzed the solid-state NMR and fluorescence experiments. K.F., K.S., and K.-i.N. prepared the MPIase. T.S. performed and analyzed the DAG fusion experiments. Y.T. and K.M. performed and analyzed the lateral diffusion experiment. K.N. and K.S. wrote and edited the manuscript with contributions from all authors.

ACKNOWLEDGMENTS

We thank Dr. Michael Moser and Dr. Maria Huber for preparing the ^{13}C-labeled natural MPIase, and Dr. Takehiko Inaba for helpful information about the flop-flop assay.

This work was supported by a Grant-in-Aid for Scientific Research (18K06143) to K.N., (17K13262) to K.F., (15KT0073 and 17H02209) to K.-i.N., and (18H04433) to K.S. from the Japan Society for the Promotion of Science and by the joint research program of Biosignal Research Center, Kobe University to K.N.

REFERENCES

1. Luirink, J., G. von Heijne, …, J. W. de Gier. 2005. Biogenesis of inner membrane proteins in *Escherichia coli*. *Annu. Rev. Microbiol.* 59:329–355.
2. Rapoport, T. A., V. Goder, …, K. E. Matlack. 2004. Membrane-protein integration and the role of the translocation channel. *Trends Cell Biol.* 14:568–575.
3. Wolfe, P. B., M. Rice, and W. Wickner. 1985. Effects of two sec genes on protein assembly into the plasma membrane of *Escherichia coli*. *J. Biol. Chem.* 260:1836–1841.
4. Chen, M., J. C. Samuelson, …, R. E. Dalbey. 2002. Direct interaction of YidC with the Sec-independent Pf3 coat protein during its membrane protein insertion. *J. Biol. Chem.* 277:7670–7675.
5. Geller, B. L., and W. Wickner. 1985. M13 procoat inserts into liposomes in the absence of other membrane proteins. *J. Biol. Chem.* 260:13281–13285.
6. Kiefer, D., and A. Kuhn. 1999. Hydrophobic forces drive spontaneous membrane insertion of the bacteriophage Pf3 coat protein without topological control. *EMBO J.* 18:6299–6306.
7. Serek, J., G. Bauer-Manz, …, A. Kuhn. 2004. *Escherichia coli* YidC is a membrane insertase for Sec-independent proteins. *EMBO J.* 23:294–301.
8. Kawashima, Y., E. Miyazaki, …, K. Nishiyama. 2008. Diacylglycerol specifically blocks spontaneous integration of membrane proteins and allows detection of a factor-assisted integration. *J. Biol. Chem.* 283:24489–24496.
9. Nishiyama, K., A. Ikegami, …, M. Müller. 2006. A derivative of lipid A is involved in signal recognition particle/SecYEG-dependent and -independent membrane integrations. *J. Biol. Chem.* 281:35667–35676.
10. Gómez-Fernández, J. C., and S. Corbalán-García. 2007. Diacylglycerols, multivalent membrane modulators. *Chem. Phys. Lipids.* 148:1–25.
11. Nishiyama, K., M. Maeda, …, H. Tokuda. 2010. A novel complete reconstitution system for membrane integration of the simplest membrane protein. *Biochem. Biophys. Res. Commun.* 394:733–736.
12. Nishiyama, K., M. Maeda, …, K. Shimamoto. 2012. MPIase is a glycolipozyme essential for membrane protein integration. *Nat. Commun.* 3:1260.
13. Fujikawa, K., S. Suzuki, …, K. Shimamoto. 2018. Syntheses and activities of the functional structures of a glycolipid essential for membrane protein integration. *ACS Chem. Biol.* 13:2719–2727.
14. Alwarawrah, M., J. Dai, and J. Huang. 2012. Modification of lipid bilayer structure by diacylglycerol: a comparative study of diacylglycerol and cholesterol. *J. Chem. Theory Comput.* 8:749–758.

15. Schorn, K., and D. Marsh. 1996. Dynamic chain conformations in dimyristoyl glycerol-dimyristoyl phosphatidylcholine mixtures. 2H-NMR studies. *Biophys. J.* 71:3320–3329.

16. Halic, M., M. Gartmann, …, R. Beckmann. 2006. Signal recognition particle receptor exposes the ribosomal translocon binding site. *Science.* 312:745–747.

17. Kaufmann, A., E. H. Manting, …, C. van der Does. 1999. Cysteine-directed cross-linking demonstrates that helix 3 of SecE is close to helix 2 of SecY and helix 3 of a neighboring SecE. *Biochemistry.* 38:9115–9125.

18. Mitra, K., C. Schaffitzel, …, J. Frank. 2005. Structure of the E. coli protein-conducting channel bound to a translating ribosome. *Nature.* 438:318–324.

19. Tanaka, Y., Y. Sugano, …, T. Tsukazaki. 2015. Crystal structures of SecYEG in lipidic cubic phase elucidate a precise resting and a peptide-bound state. *Cell Rep.* 13:1561–1568.

20. Tsukazaki, T., H. Mori, …, O. Nureki. 2008. Conformational transition of Sec machinery inferred from bacterial SecYE structures. *Nature.* 455:988–991.

21. Van den Berg, B., W. M. Clemons, Jr., …, T. A. Rapoport. 2004. X-ray structure of a protein-conducting channel. *Nature.* 427:36–44.

22. Voorhees, R. M., I. S. Fernández, …, R. S. Hegde. 2014. Structure of the mammalian ribosome-Sec61 complex to 3.4 Å resolution. *Cell.* 157:1632–1643.

23. Zimmer, J., Y. Nam, and T. A. Rapoport. 2008. Structure of a complex of the ATPase SecA and the protein-translocation channel. *Nature.* 455:936–943.

24. Matsumori, N., T. Yasuda, …, M. Murata. 2012. Comprehensive molecular motion capture for sphingomyelin by site-specific deuterium labeling. *Biochemistry.* 51:8363–8370.

25. Nomura, K., T. Inaba, …, S. Kusumoto. 2008. Interaction of lipopolysaccharide and phospholipid in mixed membranes: solid-state 31P-NMR spectroscopic and microscopic investigations. *Biophys. J.* 95:1226–1238.

26. Nieva, J. L., F. M. Goñi, and A. Alonso. 1989. Liposome fusion catalytically induced by phospholipase C. *Biochemistry.* 28:7364–7367.

27. Shimanouchi, T., H. Kawasaki, …, R. Kuboi. 2013. Membrane fusion mediated by phospholipase C under endosomal pH conditions. *Colloids Surf. B Biointerfaces.* 103:75–83.

28. Villar, A. V., F. M. Goñi, and A. Alonso. 2001. Diacylglycerol effects on phosphatidylinositol-specific phospholipase C activity and vesicle fusion. *FEBS Lett.* 494:117–120.

29. Yang, Y., H. Yao, and M. Hong. 2015. Distinguishing bicontinuous lipid cubic phases from isotropic membrane morphologies using (31) P solid-state NMR spectroscopy. *J. Phys. Chem. B.* 119:4993–5001.

30. Seelig, J. 1977. Deuterium magnetic resonance: theory and application to lipid membranes. *Q. Rev. Biophys.* 10:353–418.

31. Antollini, S. S., M. A. Soto, …, F. J. Barrantes. 1996. Physical state of bulk and protein-associated lipid in nicotinic acetylcholine receptor-rich membrane studied by laurdan generalized polarization and fluorescence energy transfer. *Biophys. J.* 70:1275–1284.

32. Dietrich, C., L. A. Bagatolli, …, E. Gratton. 2001. Lipid rafts reconstituted in model membranes. *Biophys. J.* 80:1417–1428.

33. Dinic, J., H. Biverståhl, …, I. Parmryd. 2011. Laurdan and di-4-ANEPPDHQ do not respond to membrane-inserted peptides and are good probes for lipid packing. *Biochim. Biophys. Acta.* 1808:298–306.

34. Niko, Y., P. Didier, …, A. S. Klymchenko. 2016. Bright and photostable push-pull pyrene dye visualizes lipid order variation between plasma and intracellular membranes. *Sci. Rep.* 6:18870.

35. Jay, A. G., and J. A. Hamilton. 2017. Disorder amidst membrane order: standardizing laurdan generalized polarization and membrane fluidity terms. *J. Fluoresc.* 27:243–249.

36. Jurkiewicz, P., L. Cwiklik, …, M. Hof. 2012. Lipid hydration and mobility: an interplay between fluorescence solvent relaxation experiments and molecular dynamics simulations. *Biochimie.* 94:26–32.

37. Bai, J., and R. E. Pagano. 1997. Measurement of spontaneous transfer and transbilayer movement of BODIPY-labeled lipids in lipid vesicles. *Biochemistry.* 36:8840–8848.

38. Ueda, Y., A. Makino, …, T. Kobayashi. 2013. Sphingomyelin regulates the transbilayer movement of diacylglycerol in the plasma membrane of Madin-Darby canine kidney cells. *FASEB J.* 27:3284–3297.

39. Dodd, C. E., B. R. Johnson, …, S. D. Evans. 2008. Native E. coli inner membrane incorporation in solid-supported lipid bilayer membranes. *Biointerphases.* 3:FA59.

40. Hsia, C. Y., L. Chen, …, S. Daniel. 2016. A molecularly complete planar bacterial outer membrane platform. *Sci. Rep.* 6:32715.

41. Nishikawa, H., M. Sasaki, and K. I. Nishiyama. 2017. Membrane insertion of F_0 c subunit of F_0F_1 ATPase depends on glycolipozyme MPIase and is stimulated by YidC. *Biochem. Biophys. Res. Commun.* 487:477–482.

42. Björkbom, A., T. Róg, …, J. P. Slotte. 2010. Effect of sphingomyelin headgroup size on molecular properties and interactions with cholesterol. *Biophys. J.* 99:3300–3308.

43. Gagné, J., L. Stamatatos, …, J. R. Silvius. 1985. Physical properties and surface interactions of bilayer membranes containing N-methylated phosphatidylethanolamines. *Biochemistry.* 24:4400–4408.

44. Mason, J. T., and T. J. O'Leary. 1990. Effects of headgroup methylation and acyl chain length on the volume of melting of phosphatidylethanolamines. *Biophys. J.* 58:277–281.

45. Nowacka, A., N. A. Bongartz, …, D. Topgaard. 2013. Signal intensities in ^{1}H-^{13}C CP and INEPT MAS NMR of liquid crystals. *J. Magn. Reson.* 230:165–175.

46. Carrier, D., J. B. Giziewicz, …, H. C. Jarrell. 1989. Dynamics and orientation of glycolipid headgroups by 2H-NMR: gentiobiose. *Biochim. Biophys. Acta.* 983:100–108.

47. Jarrell, H. C., A. J. Wand, …, I. C. Smith. 1987. The dependence of glyceroglycolipid orientation and dynamics on head-group structure. *Biochim. Biophys. Acta.* 897:69–82.

48. Renou, J. P., J. B. Giziewicz, …, H. C. Jarrell. 1989. Glycolipid membrane surface structure: orientation, conformation, and motion of a disaccharide headgroup. *Biochemistry.* 28:1804–1814.

49. Yamaguchi, T., Y. Sakae, …, K. Kato. 2014. Exploration of conformational spaces of high-mannose-type oligosaccharides by an NMR-validated simulation. *Angew. Chem. Int.Engl.* 53:10941–10944.

50. Alwarawrah, M., F. Hussain, and J. Huang. 2016. Alteration of lipid membrane structure and dynamics by diacylglycerols with unsaturated chains. *Biochim. Biophys. Acta.* 1858:253–263.

51. Huang, J., and G. W. Feigenson. 1999. A microscopic interaction model of maximum solubility of cholesterol in lipid bilayers. *Biophys. J.* 76:2142–2157.

52. Bezrukov, S. M. 2000. Functional consequences of lipid packing stress. *Curr. Opin. Colloid Interface Sci.* 5:237–243.

53. Murayama, T., T. Masuda, …, S. Futaki. 2017. Loosening of lipid packing promotes oligoarginine entry into cells. *Angew. Chem. Int.Engl.* 56:7644–7647.

54. Allan, D., P. Thomas, and R. H. Michell. 1978. Rapid transbilayer diffusion of 1,2-diacylglycerol and its relevance to control of membrane curvature. *Nature.* 276:289–290.

55. Ganong, B. R., and R. M. Bell. 1984. Transmembrane movement of phosphatidylglycerol and diacylglycerol sulfhydryl analogues. *Biochemistry.* 23:4977–4983.

56. Kornberg, R. D., and H. M. McConnell. 1971. Inside-outside transitions of phospholipids in vesicle membranes. *Biochemistry.* 10:1111–1120.

57. Nakano, M., M. Fukuda, …, T. Handa. 2009. Flip-flop of phospholipids in vesicles: kinetic analysis with time-resolved small-angle neutron scattering. *J. Phys. Chem. B.* 113:6745–6748.

58. Filippov, A., G. Orädd, and G. Lindblom. 2003. The effect of cholesterol on the lateral diffusion of phospholipids in oriented bilayers. *Biophys. J.* 84:3079–3086.

59. Nakamura, S., S. Suzuki, …, K. I. Nishiyama. 2018. Cholesterol blocks spontaneous insertion of membrane proteins into liposomes of phosphatidylcholine. *J. Biochem.* 163:313–319.

60. Jørgensen, K., J. H. Ipsen, …, M. J. Zuckermann. 1991. The effects of density fluctuations on the partitioning of foreign molecules into lipid bilayers: application to anaesthetics and insecticides. *Biochim. Biophys. Acta.* 1067:241–253.

61. Ratto, T. V., and M. L. Longo. 2002. Obstructed diffusion in phase-separated supported lipid bilayers: a combined atomic force microscopy and fluorescence recovery after photobleaching approach. *Biophys. J.* 83:3380–3392.

62. Saxton, M. J. 1982. Lateral diffusion in an archipelago. Effects of impermeable patches on diffusion in a cell membrane. *Biophys. J.* 39:165–173.

63. Sawasato, K., S. Suzuki, and K. I. Nishiyama. 2019. Increased expression of the bacterial glycolipid MPIase is required for efficient protein translocation across membranes in cold conditions. *J. Biol. Chem* Published online April 1, 2019. https://doi.org/10.1074/jbc.RA119.008457.

Biophysical Journal, Volume *117*

Supplemental Information

Alteration of Membrane Physicochemical Properties by Two Factors for Membrane Protein Integration

Kaoru Nomura, Toshiyuki Yamaguchi, Shoko Mori, Kohki Fujikawa, Ken-ichi Nishiyama, Toshinori Shimanouchi, Yasushi Tanimoto, Kenichi Morigaki, and Keiko Shimamoto

Supplemental Information

Alteration of Membrane Physicochemical Properties by Two Factors for Membrane Protein Integration

Kaoru Nomura, Toshiyuki Yamaguchi, Shoko Mori, Kohki Fujikawa, Ken-ichi Nishiyama, Toshinori Shimanouchi, Yasushi Tanimoto, Kenichi Morigaki, and Keiko Shimamoto

METHODS

Solid-state NMR measurement

Sample preparation

Multilamellar vesicles (MLVs) of *E. coli* polar lipid (EPL) for NMR measurements (Fig. 2a, 3 and 4b) were prepared as described previously (1-3). Designated amounts of natural MPIase, mini-MPIae-3, and/or DAG were co-solubilized with EPL in chloroform/methanol and the mixture was dried under nitrogen gas stream. After solvent evaporation under vacuum overnight, the resulting lipid film was hydrated with Tris buffer (20 mM Tris-HCl, 150 mM NaCl, pH 7.5) and vortex mixed. The suspension was freeze-thawed for ten cycles and lightly sonicated. The suspension was packed within a 4 mm NMR tube closed with the sealing-equipped cap to prevent drying (Phi Creative, Kyoto, Japan). For ^{2}H NMR measurements, we added ^{2}H-labeled DMPE (5 wt%) into the EPL liposomes. So as to mimic the molar ratio of *E. coli* membrane lipid components (DMPE/DMPG = 3/1 by weight), non-labeled DMPG (1.7 wt%) was also added. Deuterium-depleted water was used to prepare the

2-[4-(2-hydroxyethyl)piperazin-1-yl]ethanesulfonic acid (HEPES) buffer.

General measurement of solid-state NMR

All solid-state NMR measurements were carried out using a Bruker Avance III 600 spectrometer (Bruker Biospin, AG, Switzerland) equipped with a narrow-bore magnet operated at a resonance frequency of 150.13 MHz for ^{13}C, 242.94 MHz for ^{31}P, 92.12 MHz for ^{2}H, and 600.13 MHz for ^{1}H. Data were recorded using a cross polarization (CP) magic angle spinning (MAS) probe and an E-free triple-resonance probe. The MAS rate was 5 kHz for the measurement of ^{13}C CP and direct polarization (DP) MAS spectra (Fig. 3a), and ^{31}P relaxation time experiments (Fig. 3b and c). ^{31}P NMR spectra (Fig. 2a) and ^{2}H spectra (Fig. 4b) were acquired without spinning. Typical 90° pulse lengths for ^{13}C, ^{31}P, ^{2}H, and ^{1}H were 4.4, 5.0, 4.5, and 4.5 μs respectively. ^{1}H-^{13}C CP measurements were performed using a ramped (from 50% to 100%) spin-lock pulse on the ^{1}H channel and a square contact pulse on the ^{13}C channel with a 2 ms contact time. During acquisition, 51 kHz SPINAL-64 ^{1}H decoupling (4) was performed for all experiments. The ^{31}P and ^{13}C chemical shifts were externally referenced to 85% H_3PO_4 (0 ppm) and the methylene carbon of adamantane (40.48 ppm), respectively (5).

Quadrupole splitting

^{2}H spectra were obtained by use of the quadrupolar echo sequence (6) with an echo delay of 30 μs, and a recycling delay of 500 ms. The number of accumulated scans varied from 500,000 to 1000,000. All ^{2}H were processed using 500 Hz line broadening. The quadrupole splitting in a ^{2}H NMR spectra is given by the equation:

$$\Delta\nu = 3/4(e^2Qq/h)(3\cos^2\gamma\text{-}1)S_{CD} \qquad \text{(S1)},$$

where (e^2Qq/h) is the quadrupole coupling constant and γ is the angle between lipid long axis and magnetic field (6). As shown in Fig. 2a, liposome was magnetically aligned in each sample, γ was almost 90°. S_{CD} is the order parameter of CD bond (C4-^{2}H4) vector against the lipid long axis direction.

^{31}P T_1 relaxation times

^{31}P T_1 relaxation times were measured using the standard inversion recovery sequence (180°-τ-90°) (7). Delay τ varied between 1 ms and 2 s. The intensities were fit to a single-exponential function, $I = 1\text{-}2\exp(-\tau/T_1.)$.

Liposome observation

Sample preparation

Large unilamellar vesicles (LUVs) used for observing liposomes by transmission electron microscope (TEM) and dynamic light scattering (DLS) (Fig. S3) and analyzing the percentage of membrane fusion (Fig. 2b) were prepared with HEPES buffer (50 mM HEPES-KOH, 150 mM NaCl, pH 7.0) as described previously (8). Four types of LUVs composed of EPL, EPL/DAG (5 wt%), EPL/MPIase (5 wt%) or EPL/DAG (5 wt%)/MPIase (5 wt%) were prepared at a final EPL lipid concentration of 0.2 mg/ml and then transformed to 100 nm LUVs using an extruder (Avestin, Ottawa, Canada).

TEM observation

Liposomes were observed using a JEOL JEM-3100FEF transmission electron microscope (JEOL, Tokyo, Japan) with an acceleration voltage of 300 kV. An aliquot of the sample was poured onto the cell and rapidly frozen using an EM-CPC (LEICA, Wetzlar, Germany). The frozen samples were thereafter loaded into the instrument to observe the cryo-TEM image.

DLS measurement

DLS measurements were carried out at 30°C using a Nano Particle Size Analyzer (SZ-100, Horiba Scientific, Japan) with a 10 mW He-Ne laser emitting at 532 nm. Samples (600 µl) were placed in disposable polystyrene cuvettes with a 1.0-cm path length. Scattered light was detected at 90° of the incident beam using an APD detector. The hydrodynamic radius values were acquired using standard mode and calculated using the Stokes-Einstein equation.

Cobalt-calcein method

The percentage of fusion of liposome membranes was measured using the cobalt (Co^{2+})-calcein method (9). The Co^{2+}-calcein (100 mM)-containing and 2,2',2'',2'''-(ethane-1,2-diyldinitrilo)tetraacetic acid (EDTA) (100 mM)-containing liposomes were mixed at a ratio of 1:1 in 1 mM cobalt citrate solution. The fusion reaction was performed by incubation of the sample at 30 or 37 °C for 20 min. When the liposomes were fused, the fluorophore was liberated from the quenching mode by chelation of Co^{2+} with EDTA. The fluorescence intensity induced by free calcein within the fused liposomes was measured at an excitation wavelength of 490 nm and an emission wavelength of 520 nm. Fluorescence due to the leaking of liposome content was immediately quenched by cobalt citrate in the bulk solution. Without EDTA, neither the Co^{2+}-calcein complex nor calcein-citrate complex showed fluorescence. The percentage of membrane fusion was calculated from the equation:

$$\text{Fusion (\%)} = 100 * I_m/I_t, \quad \text{(S2)}$$

where I_m is the measured fluorescence intensity of the sample. The total fluorescence, I_t,

was determined after the disruption of the liposome membrane by the addition of Triton X-100 at a final concentration of 2 mM in the absence of cobalt citrate solution.

Membrane packing measurement

To prepare LUVs for lipid packing analyses (Fig. 4c and d), laurdan was added to the lipid mixture at a molar ratio of 1:100. Fluorescence spectra were acquired on a F-4500 fluorescence spectrophotometer (HITACHI High-Technologies Corp., Tokyo, Japan). The emission spectra were measured from 400 to 600 nm with an excitation wavelength of 360 nm and a 5 nm bandwidth at 30 °C. The background measured in HEPES buffer was subtracted from all emission spectra. Laurdan generalized polarization (GP) was calculated using the following equation:

$$GP = (I_{440} - I_{490})/(I_{440} + I_{490}), \qquad \text{(S3)}$$

where I_{440} and I_{490} are the intensities at 440 and 490 nm of the emission spectrum, respectively (10).

Flip-flop assay

To obtain 1-palmitoyl-2-{12-[(7-nitro-2-1,3-benzoxadiazol-4-yl)amino]dodecanoyl}-sn-glycerol (C_{12}-NBD-DAG), 1 mg of 1-palmitoyl-2-{12-[(7-nitro-2-1,3-Benzoxadiazol -4-yl)amino]dodecanoyl}-sn-glycero-3-phosphocholine (C_{12}-NBD-PC) (was digested for 2 h at 37 °C using *C. welchii*-phospholipase C (10 U) in 3.0 ml of diethyl ether/95% ethanol/water (49:1:50, v/v/v) with 0.6 μM $CaCl_2$ under shaking. Then, the produced C_{12}-NBD-DAG in the upper phase was extracted and washed with the mixture of

chloroform/water (1:1, v/v) twice. The purity of C_{12}-NBD-DAG was checked by thin-layer chromatography (TLC) and NMR. The weight of C_{12}-NBD-DAG was measured with an Ultra-Microbalance XP6V (Mettler-Toledo, Greifensee, Switzerland).

A designated amount of mini-MPIase-3 was solubilized with EPL in chloroform. C_{12}-NBD-DAG was added to EPL at a weight ratio of 1:100 and then the mixture was dried completely. The lipid film was hydrated with HEPES buffer for an EPL concentration of 0.1 mM. The suspension was freeze-thawed for ten cycles and extruded through a 100-nm pore filter (Avestin) to give LUV. To evaluate the effects of mni-MPIase-3 on the dithionite quenching in the outer leaflet, we prepared the C_{12}-NBD-PC containing liposomes in the same manner.

After monitoring the fluorescence of 350 μl LUV (100 μM) without dithionite for more than 10 s on an F-4500 fluorescence spectrophotometer (HITACHI High-Technologies Corp.) at excitation and emission wavelengths of 475 and 530 nm, respectively, 50 μl of 100 mM sodium dithionite (final concentration 12.5 mM) was added to the LUV suspension (final LUV concentration 87.5 μM). The time course of the fluorescence decrease by dithionite was continuously monitored. The flip-flop time constant (t_{flop}) was calculated from the dithionite quenching profile (11-13). The normalized fluorescence intensities F(t)/F(0), where F(0) is the fluorescence intensity just before adding dithionite, is shown by the following equation (12):

$$F(t)/F(0) = A_{inner} \exp(-t/t_{flop}) + A_{outer} \exp(-t/t_q) + C \quad \text{(S4)}$$

where A_{inner} and A_{outer} are the molar fraction of the fluorescent lipids in the inner and outer leaflets before adding dithionite, and t_{flop} and t_q are the time constants of the

flip-flop movement and dithionite quenching of C_{12}-NBD-DAG in the outer leaflet, respectively. C is the fluorescence intensity where the fluorescence attained a plateau. Usually, fluorescence was not completely quenched because some vesicles still formed multilamellar structures.

Formation of supported planar lipid bilayers

Small unilamellar vesicles (SUVs) used for supported planar lipid bilayer (SPB) observations (Fig. 6) were prepared as described previously (3). Four types of SUVs composed of EPL, EPL/DAG (5 wt%), EPL/DAG (5 wt%)/MPIase (5 wt%), and EPL/DAG (5 wt%)/MPIase (20 wt%) were prepared at a final EPL lipid concentration of 1 mg/ml in phosphate buffer (10 mM Na_2HPO_4, 150 mM NaCl, pH 6.6). Finally, the lipid dispersions were sonicated in ice water for 3-5 min using a Branson SFX150 Sonifier (Branson Ultrasonics, Danbury, CT) at 5 W. For observation of SPBs, 0.01 mg/ml of TR-DHPE was added to the EPL SUVs as a component of the lipid mixtures.

Glass slides were cleaned first by sonicating in 0.1 M SDS solution for 20 min and rinsing with deionized water. Then, they were treated in a cleaning solution of 0.05:1:5 NH_4OH (28%)/H_2O_2 (30%)/H_2O for 10 min at 65 °C and again rinsed extensively with deionized water, and then dried in a vacuum oven for 30 min at 80 °C. After cleaning by exposure to UV-produced ozone for 30 min, a SUV suspension was deposited onto a glass slide. The vesicles were allowed to adsorb and form SPBs on the surface at room temperature. SPBs thus formed were subsequently rinsed with the HEPES buffer.

Fluorescence images of SPBs were acquired with a BX51WI microscope (Olympus, Tokyo, Japan) equipped with a water immersion objective lens (60x, N.A. = 0.9, Olympus) and a 75 W Xenon lamp (LH75XEAPO). WIY filters (Olympus) were

used to detect Texas-Red fluorescence. Images of SPB fluorescence were captured with a charge coupled device (CCD) camera (DP30BW, Olympus) mounted on the microscope and further processed with Metamorph (version 6.3, Molecular Devices, CA, U.S.A.) software.

Lateral diffusion measurement by the boundary profile evolution method

Lateral diffusion coefficients of the bilayers with/without DAG and MPIase were determined by the boundary profile evolution (BPE) method (14, 15) as described previously (3). After partially photo bleaching TR-PE in the SPBs by intense illumination for 15 s, the fluorescence recovery was monitored by obtaining images every 10 s. The intensity profiles perpendicular to the boundary were nonlinearly fitted to the following Gaussian error function:

$$2\frac{F(x,t)-F_{\mathrm{bleached}}}{F_{\mathrm{unbleached}}-F_{\mathrm{bleached}}}=\mathrm{erf}\left(\frac{x-x_{\mathrm{b}}}{2w}\right)+1, \quad \text{(S5)}$$

where $F(x, t)$ is the profile evolution with time, F_{bleached} and $F_{\mathrm{unbleached}}$ are the fluorescence intensities inside and outside of the bleached region immediately after the bleaching, respectively, x_{b} is the position of the boundary between the bleached and unbleached areas, and $(x\text{-}x_{\mathrm{b}})$ is the distance to this boundary. The diffusion depth w is defined as

$$w=\sqrt{Dt}, \quad \text{(S6)}$$

where D is the diffusion coefficient and t is the elapsed time after bleaching. All measurements were conducted at room temperature.

TABLES

Table S1. Average fatty acid composition of *E.coli* phosphatidylethanolamine (PE), phosphatidylglycerol (PG), and cardiolipin(CL) in *E.coli* polar extract (16). Each component in EPL contains several types of fatty acids.

E.coli PE	
fatty acid	% of total fatty acid
18:1	34.1
16:0	33.6
17:0	17.7
16:1	9.3
19:0	3.8
14:0	1.5

E.coli PG	
fatty acid	% of total fatty acid
16:0	43.6
cyclo 17:0	26.8
Trans 18:1	9.7
16:1	7.4
cyclo 19:0	5.6
14:0	1.6
15:0	1.2
17:0	0.5
18:0	0.4
cis 18:0	0.1
unknown	3.2

E. coli CL	
fatty acid	% of total fatty acid
16:0	33.3
cyclo 17:0	27.0
trans 18:1	14.4
16:1	10.2
19:0	5.3
15:0	4.8
14:0	2.2
17:0	1.4
unknown	1.4

Table S2. Time constant of C_{12}-NBD-DAG flip-flop movement, t_{flop}, and dithionite quenching of C_{12}-NBD-DAG in the outer leaflet, t_q, in EPL/C_{12}-NBD-DAG (100:1 w/w) liposomes with various amounts of MPIase at 30 °C.

MPIase conc. (wt%)	t_{flop} (sec)	t_q(sec)
0	18.77 ± 0.03	1.74 ± 0.02
5	23.49 ± 0.04	1.76 ± 0.02
20	33.49 ± 0.10	3.26 ± 0.00
50	48.63 ± 0.22	4.44 ± 0.06

Normalized fluorescence decay curve shown in Figure 5a were fitted with the double exponential decay equation, $F(t)/F(0) = A_{inner} \exp(-t/t_{flop}) + A_{outer} \exp(-t/t_q) + C$, in Eq. S4.

Table S3. Time constant of C_{12}-NBD-DAG flip-flop movement, t_{flop}, and dithionite quenching of C_{12}-NBD-DAG in the outer leaflet, t_q, in EPL/C_{12}-NBD-DAG (100:1 w/w) liposomes with various amounts of NaCl in the presence and absence of MPIase (20 wt%) at 30 °C.

NaCl conc. (mM)	MPIase conc. (wt%)	t_{flop} (sec)	t_q(sec)
150	20	33.49 ± 0.10	3.26 ± 0.00
	0	18.77 ± 0.03	1.74 ± 0.02
300	20	28.41 ± 0.08	2.26 ± 0.03
	0	17.89 ± 0.02	1.41 ± 0.01
500	20	21.27 ± 0.03	1.51 ± 0.01
	0	18.60 ± 0.04	1.34 ± 0.01

Normalized fluorescence decay curve shown in Figure 5b were fitted with the double exponential decay equation, $F(t)/F(0) = A_{inner} \exp(-t/t_{flop}) + A_{outer} \exp(-t/t_q) + C$, in Eq. S4.

Table S4. Time constant of dithionite quenching of C_{12}-NBD-PC in the outer leaflet, t_q, in EPL/C_{12}-NBD-PC (100:1 w/w) liposomes with various amounts of MPIase at 30 °C.

MPIase conc. (wt%)	t_q(sec)
0	10.86 ± 0.05
5	11.49 ± 0.04
20	22.13 ± 0.04

Normalized fluorescence decay curve shown were fitted with the single exponential decay equation, $F(t)/F(0) = A_{outer} \exp(-t/t_q) + C$.

DATA GRAPHS

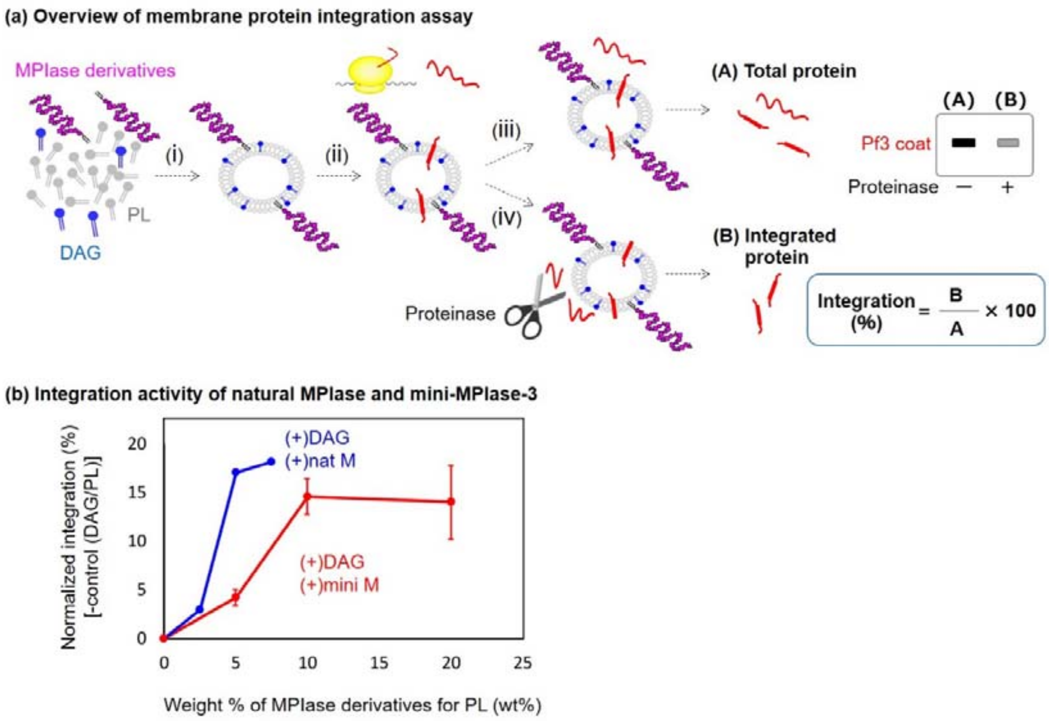

Figure S1. (a) Overview of the membrane protein integration activity assay. (i) Liposomes are prepared from DAG (5 wt%), *E. coli* polar lipids (EPL) and MPIase derivatives. (ii) Radioisotope-labeled 3L-Pf3 proteins are synthesized using an *in vitro* translation system in the presence of liposomes. (iii) The reaction mixture is divided into two fractions: One fraction gives the total amount of synthesized proteins by autoradiography after SDS-PAGE (band A). (iv) When protease is added to the other fraction, proteins outside liposomes are cleaved; thus, band B reflects the amount of proteins integrated in the liposomes. (b) Dose-response of the integration activity by MPIase derivatives. Normalized integration values were calculated by the subtraction of the percentage of control liposomes containing only DAG from the net integration percentages of liposomes containing MPIase derivatives.

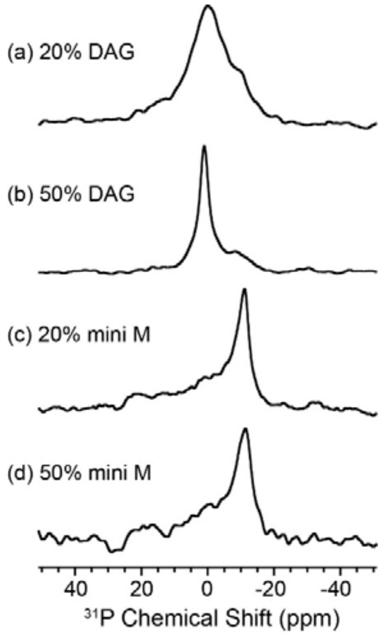

Figure S2. ^{31}P NMR spectra of *E. coli* polar lipid (EPL) in the presence of 20 and 50 wt% DAG (a and b) or 20 and 50 wt% mini-MPIase-3 (c and d). The amounts of DAG and mini-MPIase-3 are shown against the amount of EPL.

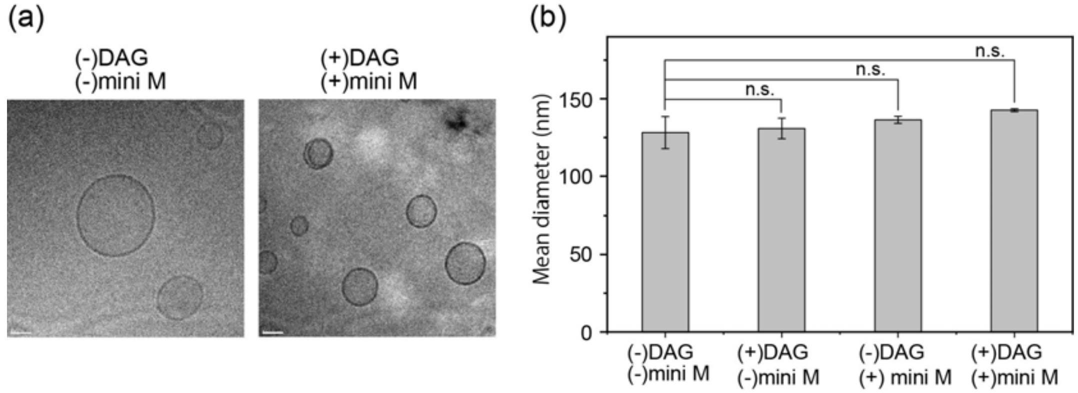

Figure S3. TEM image of EPL liposomes in the absence (left) and presence of DAG (5 wt%)/mini-MPIase-3 (5 wt%) (right) at 30 °C. Scale bar = 50 nm. (b) The average vesicle size of EPL liposome in the absence and presence of DAG and/or mini-MPIase-3 (Fig. S3b) determined by DLS. The error bars show the standard deviation of three experiments.

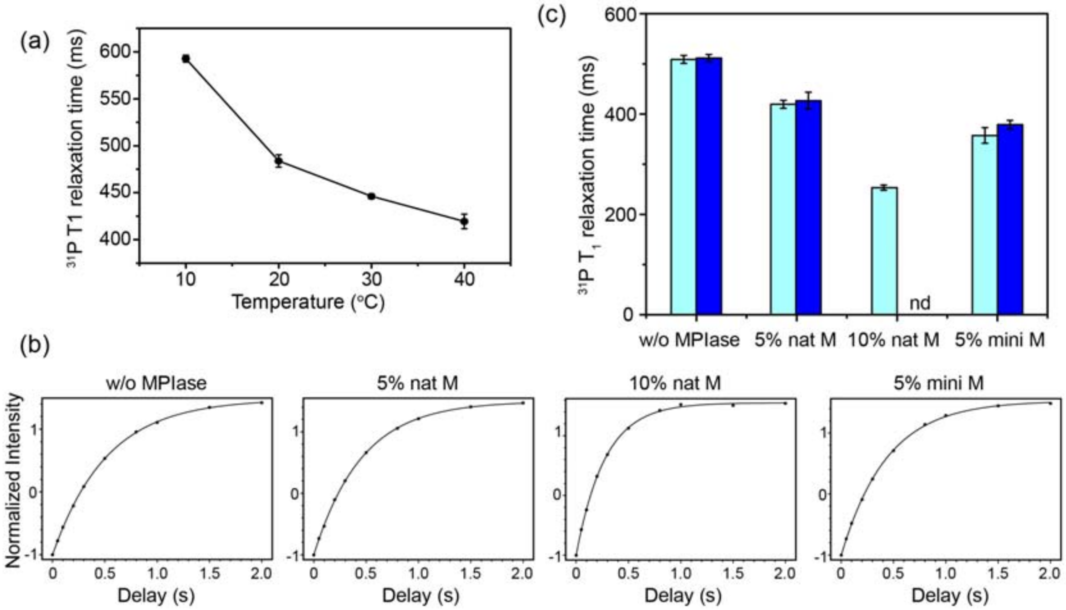

Figure S4. (a) ^{31}P T_1 relaxation times of EPL liposomes in the presence of natural MPIase (5 wt%) as a function of temperature. Decrease of T_1 with increasing temperature indicates that the longer T_1 means slower motion in this sample. (b) Representative normalized signal intensity curves in the inversion recovery experiment of EPL liposomes in the absence and presence of natural MPIase (5 wt%), natural MPIase (10 wt%), and mini-MPIase-3 (5 wt%) at 30 °C. (c) Effects of DAG, mini-MPIase-3 and natural MPIase on the ^{31}P T_1 values of EPL liposomes at 40 °C. From the left, the results of ^{31}P T_1 values in the following conditions are shown in the presence (light blue) and absence (blue) of DAG: without MPIase, natural MPIase (5 wt%), natural MPIase (10 wt%), and mini-MPIase-3 (5 wt%).

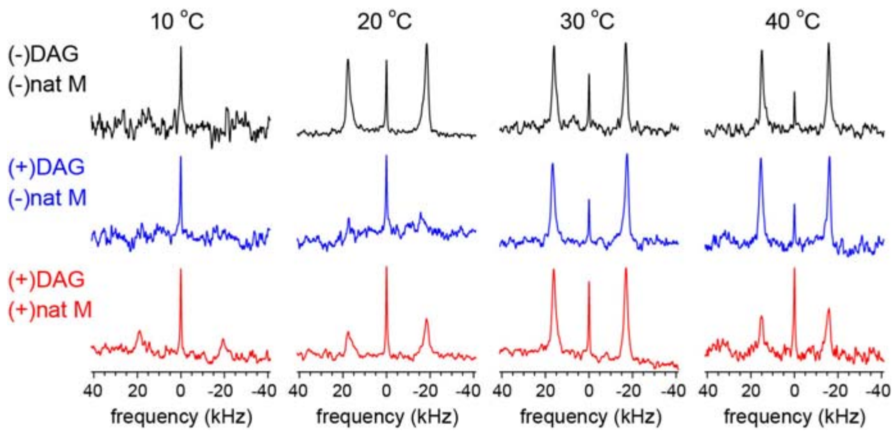

Figure S5. The quadrupole splitting of ^{2}H spectra at the designated temperature in the absence (black) and presence of DAG (5 wt%) (blue), and DAG (5 wt%)/natural MPIase (5 wt%) (red).

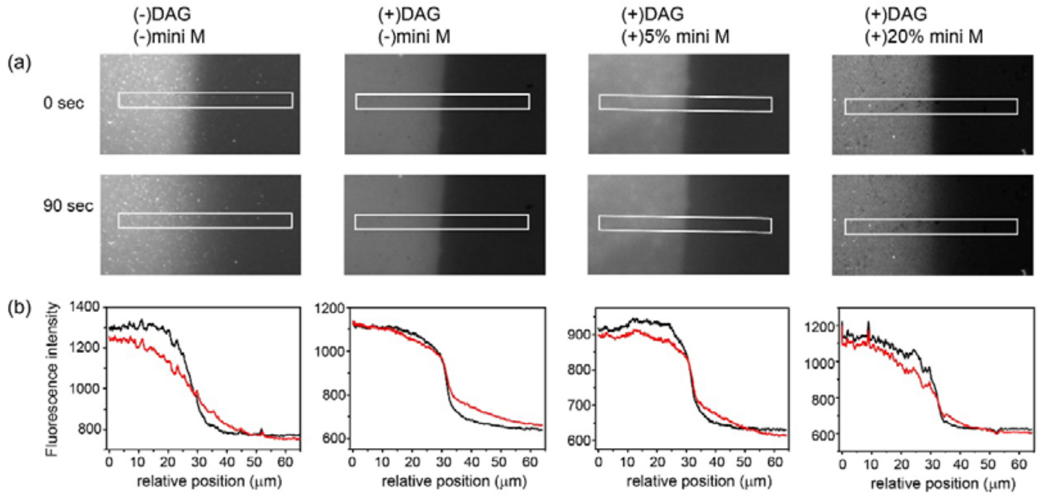

Figure S6. FRAP images for TR-DHPE in EPL SPBs in the absence and presence of DAG (5 wt%), DAG (5 wt%)/mini-MPIase-3 (5 wt%), or DAG (5 wt%)/mini-MPIase-3 (20 wt%) at 30 °C. The left half of the images was bleached, and images were taken immediately after bleaching (0 sec after bleaching) and 90 sec after bleaching. (b) Fluorescence intensity profile for a 65 μm stripe in *x* across the bleached region as shown in (a) at 0 (black) and 90 (red) sec after bleaching.

REFERENCES

S1. Nomura, K., G. Corzo, T. Nakajima, and T. Iwashita. 2004. Orientation and pore-forming mechanism of a scorpion pore-forming peptide bound to magnetically oriented lipid bilayers. Biophys. J. 87:2497-2507.

S2. Nomura, K., G. Ferrat, T. Nakajima, H. Darbon, T. Iwashita, and G. Corzo. 2005. Induction of morphological changes in model lipid membranes and the mechanism of membrane disruption by a large scorpion-derived pore-forming peptide. Biophys. J. 89:4067-4080.

S3. Nomura, K., T. Inaba, K. Morigaki, K. Brandenburg, U. Seydel, and S. Kusumoto. 2008. Interaction of lipopolysaccharide and phospholipid in mixed membranes: solid-state 31P-NMR spectroscopic and microscopic investigations. Biophys. J. 95:1226-1238.

S4. Fung, B. M., A. F. Khitrin, and K. Ermolaev. 2000. An improved broadband decoupling sequence for liquid crystals and solids. J. Magn. Reson. 142:97-101.

S5. Morcombe, C. R., and K. W. Zilm. 2003. Chemical shift referencing in MAS solid state NMR. J. Magn. Reson. 162:479-486.

S6. Seelig, J. 1977. Deuterium magnetic resonance: theory and application to lipid membranes. Q. Rev. Biophys. 10:353-418.

S7. Vold, R. L., J. S. Waugh, M. P. Klein, and D. E. Phelps. 1968. Measurement of Spin Relaxation in Complex Systems. J. Chem. Phys. 48:3831.

S8. Nomura, K., Y. Tanimoto, F. Hayashi, E. Harada, X. Y. Shan, M. Shionyu, A. Hijikata, T. Shirai, K. Morigaki, and K. Shimamoto. 2017. The Role of the Prod1 Membrane Anchor in Newt Limb Regeneration. Angew. Chem. Int. Ed. Engl. 56:270-274.

S9. Shimanouchi, T., H. Kawasaki, M. Fuse, H. Umakoshi, and R. Kuboi. 2013. Membrane fusion mediated by phospholipase C under endosomal pH conditions. Colloids and surfaces. B, Biointerfaces 103:75-83.

S10. Parasassi, T., G. De Stasio, A. d'Ubaldo, and E. Gratton. 1990. Phase fluctuation in phospholipid membranes revealed by Laurdan fluorescence. Biophys. J. 57:1179-1186.

S11. Bai, J., and R. E. Pagano. 1997. Measurement of spontaneous transfer and transbilayer movement of BODIPY-labeled lipids in lipid vesicles. Biochemistry 36:8840-8848.

S12. Nakao, H., K. Ikeda, Y. Ishihama, and M. Nakano. 2016. Membrane-Spanning Sequences in Endoplasmic Reticulum Proteins Promote Phospholipid Flip-Flop.

Biophys. J. 110:2689-2697.

S13. Ueda, Y., A. Makino, K. Murase-Tamada, S. Sakai, T. Inaba, F. Hullin-Matsuda, and T. Kobayashi. 2017. Sphingomyelin regulates the transbilayer movement of diacylglycerol in the plasma membrane of Madin-Darby canine kidney cells. FASEB J. 27:3284-3297.

S14. Merzlyakov, M., E. Li, and K. Hristova. 2006. Directed assembly of surface-supported bilayers with transmembrane helices. Langmuir 22:1247-1253.

S15. Okazaki, T., T. Inaba, Y. Tatsu, R. Tero, T. Urisu, and K. Morigaki. 2009. Polymerized lipid bilayers on a solid substrate: morphologies and obstruction of lateral diffusion. Langmuir 25:345-351.

S16. website of Avanti Polar Lpids : https://avantilipids.com/.
